# Pericellular Ca^2+^ recycling potentiates thrombin-evoked Ca^2+^ signals in human platelets

**DOI:** 10.1002/phy2.85

**Published:** 2013-10-11

**Authors:** Stewart O Sage, Nicholas Pugh, Richard W Farndale, Alan G S Harper

**Affiliations:** 1 Department of Physiology, Development and Neuroscience, University of CambridgeCambridge, U.K; 2 Department of Biochemistry, University of CambridgeCambridge, U.K; 3 Institute for Science and Technology in Medicine, Keele UniversityStoke-on-Trent, U.K

**Keywords:** Ca^2+^, FFP-18, nanojunction, open canalicular system, pericellular, platelets

## Abstract

We have previously demonstrated that Na^+^/Ca^2+^ exchangers (NCXs) potentiate Ca^2+^ signaling evoked by thapsigargin in human platelets, via their ability to modulate the secretion of autocoids from dense granules. This link was confirmed in platelets stimulated with the physiological agonist, thrombin, and experiments were performed to examine how Ca^2+^ removal by the NCX modulates platelet dense granule secretion. In cells loaded with the near-membrane indicator FFP-18, thrombin stimulation was observed to elicit an NCX-dependent accumulation of Ca^2+^ in a pericellular region around the platelets. To test whether this pericellular Ca^2+^ accumulation might be responsible for the influence of NCXs over platelet function, platelets were exposed to fast Ca^2+^ chelators or had their glycocalyx removed. Both manipulations of the pericellular Ca^2+^ rise reduced thrombin-evoked Ca^2+^ signals and dense granule secretion. Blocking Ca^2+^-permeable ion channels had a similar effect, suggesting that Ca^2+^ exported into the pericellular region is able to recycle back into the platelet cytosol. Single cell imaging with extracellular Fluo-4 indicated that thrombin-evoked rises in extracellular [Ca^2+^] occurred within the boundary described by the cell surface, suggesting their presence within the open canalicular system (OCS). FFP-18 fluorescence was similarly distributed. These data suggest that upon thrombin stimulation, NCX activity creates a rise in [Ca^2+^] within the pericellular region of the platelet from where it recycles back into the platelet cytosol, acting to both accelerate dense granule secretion and maintain the initial rise in cytosolic [Ca^2+^].

## Introduction

The importance of rises in cytosolic Ca^2+^ concentration ([Ca^2+^]_cyt_) in the activation of human platelets is well established (Rink and Sage 1990). This has led to great interest in identifying the molecular mechanisms that generate and modulate agonist-evoked rises in platelet [Ca^2+^]_cyt_, in the hope of identifying novel targets for antithrombotic drugs. Most experiments have examined the effects of pharmacological, molecular or physiological manipulations on agonist-evoked rises in [Ca^2+^]_cyt_ alone. However, our recent work has demonstrated that conclusions as to how the platelet Ca^2+^ signaling system is affected by such manipulations when measuring agonist-evoked rises in [Ca^2+^]_cyt_ alone are frequently erroneous (Harper and Sage 2007; Harper et al. 2009b, 2010; Sage et al. 2011). By additionally examining the effects of experimental manipulations on agonist-evoked changes in several variables that influence intracellular Ca^2+^ signaling, including the divalent cation permeability of the plasma membrane, the cytosolic Na^+^ concentration ([Na^+^]_cyt_), the Ca^2+^ concentration in intracellular stores ([Ca^2+^]_st_), the extracellular Ca^2+^ concentration ([Ca^2+^]_ext_), the pericellular Ca^2+^ concentration ([Ca^2+^]_peri_) and the secretion of autocrine activators from dense granules, we have created an outline of how the platelet Ca^2+^ signaling system functions at a systems level. Using this information we have been able to refine our understanding of how platelet Ca^2+^ signaling is affected by a variety of experimental interventions.

Recently, we have been attempting to understand how the Na^+^/Ca^2+^ exchanger (NCX) influences platelet Ca^2+^ signaling and thus platelet function. NCX3 has been shown to be the predominant isoform of this exchanger expressed in human platelets in both a proteomic screen of the platelet plasma membrane (Lewandrowski et al. 2009) and by Western blotting (Harper et al. 2010; Roberts et al. 2012). Through the use of both pharmacological inhibitors of the NCX and physiological inhibition of this exchanger by replacement of extracellular Na^+^ with *N*-methyl-d-glucamine (NMDG), we and others have demonstrated that the NCX plays a key role in regulating rises in [Ca^2+^]_cyt_ evoked by a variety of agonists (Roberts et al. 2004; Harper and Sage 2007; Harper et al. 2009b, 2010). Our previous work has demonstrated an important role for the NCX in potentiating store-operated and P_2x1_-mediated Ca^2+^ entry in human platelets (Harper and Sage 2007; Harper et al. 2009b, 2010). These earlier studies demonstrated that the pharmacological inhibitors of the NCX used had no direct effect on the activation of either the P_2X1_ receptor or the store-operated channel (SOC), but instead influenced the agonist-evoked rise in [Ca^2+^]_cyt_ at a point downstream of the activation of these ion channels. By analysis of a number of the factors that influence platelet Ca^2+^ signal generation, it was concluded that the NCX exerted its influence by regulating the rate of autocoid secretion from dense granules (Harper et al. 2009b). We found that the Ca^2+^ signaling defect in NCX-inhibited platelets could be reversed by exogenous addition of the autocrine signaling molecules present in dense granules (ATP, ADP, and serotonin) or by addition of releasate from uninhibited, activated platelets (Harper et al. 2009b). These data further demonstrated that the effects of pharmacological inhibitors of the NCX were not due to nonspecific block of any component of the platelet Ca^2+^ signaling apparatus.

Although these results provided an interesting insight into the role that NCX3 plays in regulating human platelet function, our earlier experiments principally used nonphysiological agonists to isolate specific Ca^2+^ signaling pathways. Here, we have used the physiological platelet agonist, thrombin, to investigate whether a link between NCX activity, dense granule secretion, and platelet Ca^2+^ signaling exists under physiological conditions. These experiments also attempted to refine our understanding of how NCX activity regulates platelet dense granule secretion. We present evidence that Ca^2+^ removal from the platelet cytosol via the NCX (forward-mode operation) creates a pericellular Ca^2+^ source from which Ca^2+^ is able to recycle back into the platelet cytosol down its concentration gradient through Ca^2+^-permeable ion channels. Furthermore, we demonstrate that experimental interference with the thrombin-evoked pericellular Ca^2+^ signal inhibits both platelet dense granule secretion and thrombin-evoked rises in [Ca^2+^]_cyt_. These observations provide new insights into how the NCX influences platelet Ca^2+^ signaling and dense granule secretion in human platelets.

## Materials and Methods

### Ethical approval

This study was approved by the University of Cambridge Human Biology Research Ethics Committee. Blood was donated by healthy, drug-free volunteers who gave written informed consent. The experiments conformed to the guidelines stated in the Declaration of Helsinki.

### Materials

5-5′-Dimethyl-BAPTA K^+^ salt was from Cambridge Biosciences (Cambridge, U.K.). FFP-18 K^+^ salt (also known as Fura-2 NM K^+^ salt), Fura-2/AM and SBFI/AM were from TEFlabs Inc. (Austin, TX). Thrombin was from Merck Chemicals (Nottingham, U.K.). Fluo-5N/AM and K^+^ salts of BAPTA and Fluo-4 were from Invitrogen (Paisley, U.K.). KB-R7943, MRS-1845, and SN-6 were from Tocris Bioscience (Bristol, U.K.). Apyrase, luciferin–luciferase, NMDG, and RGDS peptide were from Sigma Aldrich (Gillingham, U.K.). 5′-Iodo-resiniferatoxin was from LC laboratories (Woburn, MA). All other reagents were of analytical grade.

### Platelet preparation

Blood was collected by venepuncture and mixed with one-sixth volume of acid citrate dextrose anticoagulant (ACD; 85 mmol L^−1^ sodium citrate, 78 mmol L^−1^ citric acid, and 111 mmol L^−1^ D-glucose) and platelet-rich plasma (PRP) prepared by centrifugation for 5 min at 700 *g*, before aspirin (100 μmol L^−1^) and apyrase (40 μg mL^−1^) were added.

### Monitoring cytosolic Ca^2+^ concentration

Platelet-rich plasma was incubated with 2 μmol L^−1^ Fura-2/AM for 45 min at 37°C. Platelets were collected by centrifugation at 350 *g* for 20 min and resuspended in Hepes-buffered saline (HBS; 145 mmol L^−1^ NaCl, 10 mmol L^−1^ Hepes (*N*-2-hydroxyethylpiperazine-*N*’-2-ethanesulfonic acid), 10 mmol L^−1^ d-glucose, 5 mmol L^−1^ KCl, 1 mmol L^−1^ MgSO_4_, pH 7.45) supplemented with 0.1% w/v bovine serum albumin, 200 μmol L^−1^ CaCl_2_ and 40 μg mL^−1^ apyrase (supplemented HBS). Fluorescence was recorded from 1 mL stirred aliquots of platelet suspension at 37°C using a Cairn Research Spectrophotometer (Cairn Research, Faversham, U.K.) with excitation at 340 and 380 nm and emission at 515 nm. Changes in [Ca^2+^]_cyt_ were monitored using the 340/380 nm fluorescence ratio and calibrated according to the method of Grynkiewicz et al. (1985).

### Monitoring cytosolic Na^+^ concentration

Platelets were collected from PRP by centrifugation at 350 *g* for 20 min and resuspended in supplemented HBS. SBFI/AM (20 μg) was mixed with 10% pluronic F-127 dissolved in dimethylsulfoxide (DMSO) to give a stock solution of 5 mmol L^−1^. This was added to washed platelet suspensions to give a final concentration of 10 μmol L^−1^ SBFI/AM. After incubation at 37°C for 40 min, 10% v/v ACD was added and the cells were recollected by centrifugation in a microcentrifuge at 8000 *g* for 30 sec. The platelets were then resuspended in supplemented HBS and SBFI fluorescence measurements were made as for Fura-2 above. Changes in [Na^+^]_cyt_ were monitored using the SBFI 340/380 nm fluorescence ratio. We have previously described a small quenching effect of KB-R7943 on SBFI fluorescence (Harper and Sage 2007). To compensate for this, records were normalized to the basal fluorescence level before thrombin addition. However, after this normalization any Na^+^ rises in KB-R7943-treated platelets will be slightly overestimated and so any inhibition slightly underestimated.

### Monitoring intracellular store Ca^2+^ concentration

[Ca^2+^]_st_ was monitored using Fluo-5N (Sage et al. 2011). Platelet-rich plasma was incubated with 250 nmol L^−1^ Fluo-5N/AM for 2 h at 37°C. Cells were then collected by centrifugation at 350 *g* for 20 min and resuspended in supplemented HBS to which 100 μmol L^−1^ RGDS peptide was also added. RGDS was included in all experiments with Fluo-5N-loaded platelets to prevent aggregation and therefore artifactual drops in Fluo-5N fluorescence. RGDS at this concentration has previously been demonstrated not to affect [Ca^2+^]_cyt_ signals in human platelets (Rosado et al. 2001). Fura-5N fluorescence was recorded as for Fura-2 above but with an excitation wavelength of 485 nm and collecting emitted light of wavelengths between 515 and 565 nm.

### Monitoring extracellular Ca^2+^ concentration

The release of Ca^2+^ to the extracellular medium in the absence of extracellular Ca^2+^ was monitored by addition of 2.5 μmol L^−1^ Fluo-3 or Fluo-4 K^+^ salts to washed platelet suspensions immediately prior to the start of experiments. Fluorescence was monitored as for Fluo-5N.

In some experiments, Fluo-4 data were calibrated by measuring the fluorescence of a cell-free aliquot of supplemented HBS to which 2.5 μmol L^−1^ Fluo-4 K^+^ salt was added. The fluorescence of this sample was monitored as known amounts of CaCl_2_ were added to give final concentrations between 0.1 and 300 μmol L^−1^ calcium. The fluorescence at each Ca^2+^ concentration was measured and used to construct a calibration curve in which the known Ca^2+^ concentration was plotted against the measured fluorescence value using GraphPad Prism™ software (GraphPad Software, San Diego, CA). This was then subject to nonlinear regression to fit to a one-phase association equation (*R*^2^ between 0.96 and 0.99). The derived equation was then used to calibrate the Fluo-4 data.

The release of Ca^2+^ to the extracellular medium in the presence of 300 μmol L^−1^ extracellular Ca^2+^ was monitored by addition of 5 μmol L^−1^ Rhod-5N to washed platelet suspensions immediately prior to the start of experiments. Fluorescence was monitored as for Fura-2 but with excitation at 550 nm and collecting emitted light of wavelengths between 570 and 640 nm.

### Monitoring pericellular Ca^2+^ concentration

[Ca^2+^]_peri_ was monitored by treating washed platelets with 5 μmol L^−1^ FFP-18 salt for 5 min at room temperature. Cells were then separated into 1-mL aliquots to which 250 μL of ACD was added. Excess dye was removed by centrifugation for 30 sec at 8000 *g*. The supernatant was removed and the pelleted cells were resuspended in supplemented HBS. Fluorescence was recorded as for Fura-2 above. Changes in [Ca^2+^]_peri_ were monitored using the 340/380 nm fluorescence ratio. FFP-18 loading was designed to specifically label the extracellular membrane with the indicator (see Results).

In some experiments, FFP-18 data were calibrated in situ in intact FFP-18-loaded cells. Unstimulated FFP-18-loaded cells were exposed to saturating Ca^2+^ concentrations by addition of 3 mmol L^−1^ CaCl_2_ to the extracellular medium; 10 mmol L^−1^ EGTA and 20 mmol L^−1^ Tris were then added to the medium and the 340/380 nm fluorescence ratio was monitored until successive additions of EGTA and Tris elicited no further fall in the 340/380 nm ratio. The maximum and minimum 340/380 nm fluorescence ratios and the maximum and minimum values of fluorescence measured at 380 nm were then used in the same equation used by Grynkiewicz et al. (1985), along with the published K_d_ for FFP-18 of 400 nmol L^−1^ (Etter et al. 1996).

For imaging of single FFP-18-loaded cells, washed platelets were treated with 25 μmol L^−1^ FFP-18 salt for 10 min at room temperature. Excess dye was removed by centrifugation for 30 sec at 8000 *g* after the addition of 25% (v/v) ACD to the cell suspension. The supernatant was removed and the pelleted cells were resuspended in supplemented HBS. Platelets were then treated with 1 mmol L^−1^ EGTA to chelate extracellular calcium and allowed to adhere to poly-l-lysine-coated coverslips before another coverslip was placed on top to permit the use of a water immersion lens without disturbing the cells. FFP-18 fluorescence was monitored using a Leica SP-5 confocal microscope with an excitation wavelength of 405 nm and emission wavelengths of 420–580 nm.

### Quantification of thrombin-evoked changes in [Ca^2+^]_cyt_, [Ca^2+^]_ext_, [Ca^2+^]_peri_, [Ca^2+^]_st_, and [Na^+^]_cyt_

[Ca^2+^]_cyt_, [Ca^2+^]_ext_, [Ca^2+^]_peri_, [Ca^2+^]_st_*,* and [Na^+^]_cyt_ were quantified by integration of the change in fluorescence records from basal with respect to time for 3 min after thrombin addition unless stated.

### Single platelet imaging of extracellular Ca^2+^ signals

Net Ca^2+^ removal across the plasma membrane of single platelets was recorded using an FV300 laser-scanning confocal microscope (Olympus, U.K.) with a PLAPON 60× oil immersion objective and a 300-μm confocal aperture. Chambered coverslips (Nunc) were coated with collagen-related peptide (CRP; 10 μg mL^−1^) or fibrinogen (10 μg mL^−1^) overnight at 4°C. Slides were washed with Ca^2+^-free Tyrodes solution and mounted on the microscope stage. Platelets at a density of 1 × 10^8^ mL^−1^ in a Ca^2+^-free Tyrodes solution containing 2.5 μmol L^−1^ Fluo-4 were pipetted into the chambered coverslip and allowed to adhere to the substrate for 5 min. Images were recorded at a frequency of 0.9 Hz for 5 min with excitation at 488 nm and emission at 510–530 nm.

### Dense granule secretion

Luciferin–luciferase (1% [v/v] final concentration) was added to 1.5 mL stirred aliquots of platelets prior to the start of experiments. ATP secretion was measured by monitoring the light emitted from the sample in a light-protected cuvette holder connected to a high gain photomultiplier tube. Measurements of ATP secretion were taken as the integral over basal light levels (average light emitted 30 sec before stimulation) for 20 sec after stimulation. KB-R7943 (50 μmol L^−1^) and SN-6 (50 μmol L^−1^) had no detectible effect on the assay.

### Statistical analysis

Values stated are mean ± SEM of the number of observations (*n*) indicated. Analysis of statistical significance was performed using Student's *t*-test. *P* < 0.05 was considered significant.

## Results

### NCX inhibition reduces thrombin-evoked Ca^2+^ signaling in the presence and absence of extracellular Ca^2+^

**Figure 1:**
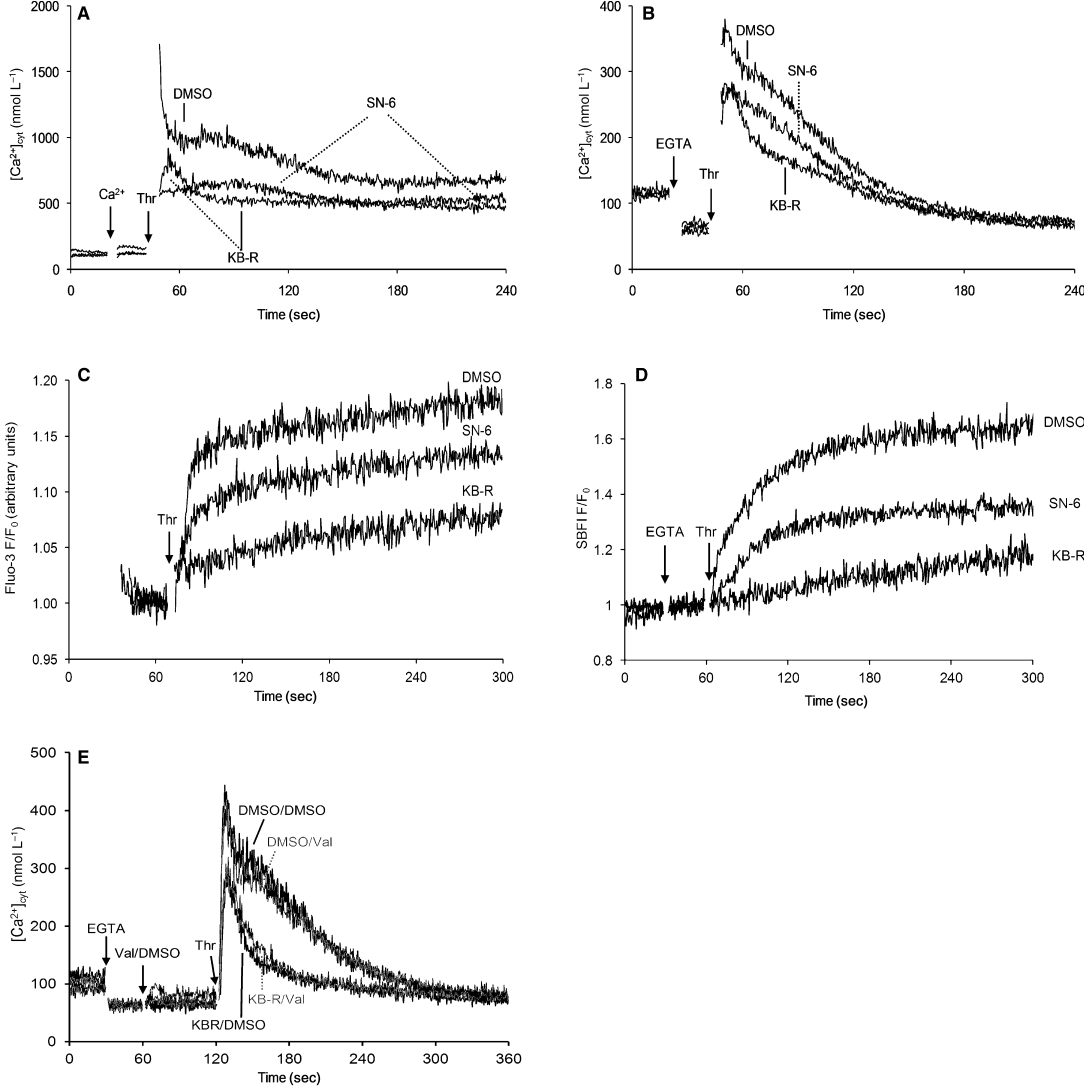
Na^+^/Ca^2+^ exchanger inhibition reduces thrombin-evoked Ca^2+^ signalling through the loss of forward mode exchange. Fura-2- (A, B, E) or SBFI-loaded human platelets (D) or platelets suspended in supplemented Hepes-buffered saline with 2.5 μmol L^−1^ Fluo-3 (C) were pre-treated with 50 μmol L^−1^ KB-R7943, 50 μmol L^−1^ SN-6 or their vehicle, dimethylsulfoxide (DMSO), for 2 min at 37°C. CaCl2 (800 μmol L^−1^; A) or EGTA (1 mmol L^−1^; B–E) were added before the cells were stimulated with 0.5 U mL^−1^ thrombin. In E, 3 μmol L^−1^ valinomycin or its vehicle, DMSO, was added 1 min prior to the addition of thrombin. In C, the addition of EGTA to the cells at 30 sec causes a rapid fall in Fluo-3 fluorescence to a much lower basal level. The data collected before EGTA addition has been omitted from the trace to ensure that thrombin-evoked rises in [Ca^2+^]ext can be seen clearly. Results presented are representative of 5–6 experiments.

The effects of the structurally distinct NCX inhibitors SN-6 and KB-R7943 on thrombin-evoked changes in [Ca^2+^]_cyt_ were investigated, using the inhibitors at concentrations previously shown to have maximal effects on store-operated Ca^2+^ entry (SOCE) in human platelets without directly affecting the Ca^2+^ signaling apparatus of these cells (Harper and Sage 2007; Harper et al. 2009b). Thrombin-evoked rises in [Ca^2+^]_cyt_ in the presence of extracellular Ca^2+^ were reduced to 64.7 ± 12.6% or 53.2 ± 10.7% of control after treatment with SN-6 (50 μmol L^−1^) or KB-R7943 (50 μmol L^−1^), respectively (both *n* = 5, *P* < 0.05; Fig. 1A). If the role of the NCX in Ca^2+^ signal generation was through reverse-mode exchange, then the NCX inhibitors should have no effect when platelets are activated in the absence of extracellular Ca^2+^. However, the NCX inhibitors also reduced thrombin-evoked rises in [Ca^2+^]_cyt_ in the absence of extracellular Ca^2+^ (65.0 ± 5.3% or 48.9 ± 8.9% of control after treatment with 50 μmol L^−1^ SN-6 or KB-R7943, respectively; both *n* = 5, *P* < 0.05; Fig. 1B). These results agree with previous studies investigating the effects of other, structurally distinct NCX inhibitors such as 3′,4′ dichlorobenzamil, Ni^2+^ and bepridil on thrombin-evoked Ca^2+^ signaling (Hunyady et al. 1987; Jy and Haynes 1987). Although these agents may have additional targets, the fact that a number of structurally unrelated NCX inhibitors all have similar effects on thrombin-evoked Ca^2+^ signals in the absence of extracellular Ca^2+^ suggests that the NCX plays a key role in generating these signals at least partially through forward-mode exchange (Hunyady et al. 1987; Jy and Haynes 1987; Harper and Sage 2007; Harper et al. 2009b, 2010).

### NCX inhibitors slow forward-mode activity of the NCX

To confirm that the effect of NCX inhibitors on thrombin-evoked rises in [Ca^2+^]_cyt_ was due to inhibition of net forward-mode NCX transport, the effects of these inhibitors on [Ca^2+^]_ext_ were investigated. Pretreatment of platelets with 50 μmol L^−1^ SN-6 or KB-R7943 in the absence of extracellular Ca^2+^ reduced thrombin-evoked rises in [Ca^2+^]_ext_ to 77.4 ± 9.7% or 26.3 ± 5.9% of control, respectively (both *n* = 6, *P* < 0.05; Fig. 1C). Pretreatment of platelets with SN-6 or KB-R7943 in the absence of extracellular Ca^2+^ also inhibited thrombin-evoked rises in [Na^+^]_cyt_ (66.3 ± 7.1% or 14.9 ± 2.8% of control, respectively; both *n* = 6, *P* < 0.05; Fig. 1D). These results are consistent with the NCX inhibitors reducing forward-mode NCX activity. Interestingly, KB-R7943 was more effective than SN-6 at inhibiting thrombin-evoked rises in [Ca^2+^]_cyt_, [Ca^2+^]_ext_, and [Na^+^]_cyt_. These differences in the effects of the two NCX inhibitors are in agreement with our previous data on the inhibition of store-operated and P_2X1_-mediated Ca^2+^ entry by these compounds (Harper and Sage 2007; Harper et al. 2010), as well as previous work showing that KB-R7943 is a more effective inhibitor of NCX3 than SN-6 (Iwamoto and Shigekawa 1998).

The directionality of NCX activity depends on the electrochemical gradients for Na^+^ and Ca^2+^ as well as on the membrane potential, parameters that change during platelet stimulation. However, thrombin is reported to evoke either no change in platelet membrane potential (MacIntyre and Rink 1982) or only a small (6–8) mV depolarization (Pipili 1985). The relatively stable membrane potential during platelet stimulation likely reflects the presence of both voltage-gated and Ca^2+^-activated K^+^ channels in these cells (Mahaut-Smith et al. 1990; Mahaut-Smith 1995). To investigate the possible influence of changes in membrane potential, the effects of KB-R7943 (50 μmol L^−1^) on the thrombin-evoked change in [Ca^2+^]_cyt_ in the absence of extracellular Ca^2+^ were investigated in the presence and absence of valinomycin (3 μmol L^−1^), which clamps the platelet membrane potential near the reversal potential of K^+^ (Mahaut-Smith et al. 1990). KB-R7943 reduced the thrombin-evoked Ca^2+^ signal to 51.0 ± 7.7% of control in the absence of valinomycin and 64.5 ± 8.4% of control in its presence (both *n* = 6, *P* < 0.05; Fig. 1E). This result indicates that membrane potential changes have little influence on the directionality of the NCX under these conditions.

Although we cannot exclude operation of the NCX in both forward and reverse mode at different times or in different subcellular locations during the stimulation of platelets with thrombin, our results are consistent with net operation of the NCX in forward mode.

### Removal of extracellular Na^+^ has similar effects to NCX inhibition

**Figure 2:**
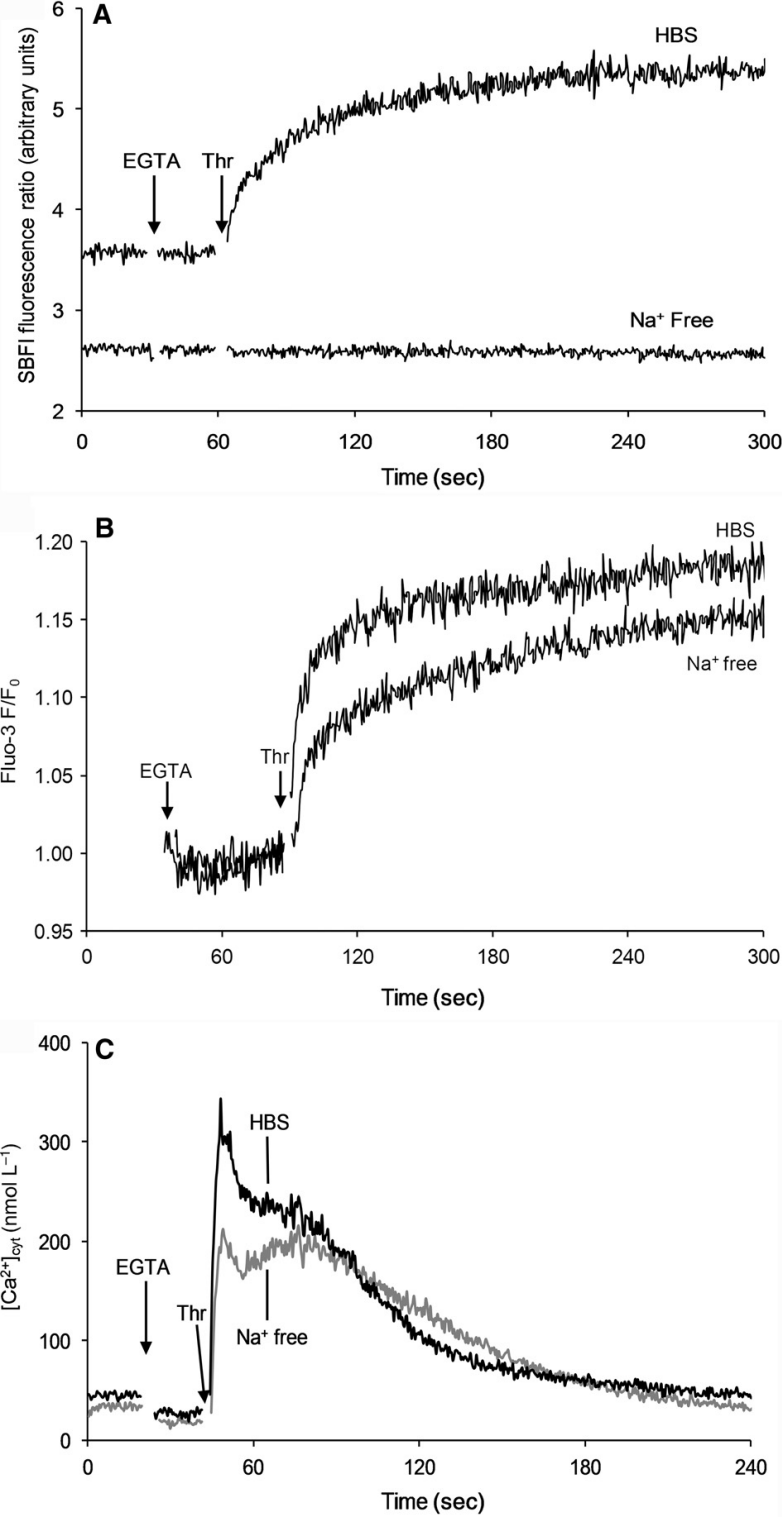
Replacement of extracellular Na^+^ has similar effects to Na^+^/Ca^2+^ exchanger inhibition on thrombin-evoked changes in [Ca^2+^]cyt. SBFI- (A) or Fura-2- (C) loaded human platelets or platelets in the presence of 2.5 μmol L^−1^ Fluo-3 (B) were suspended in supplemented Hepes-buffered saline or a medium in which extracellular Na^+^ was replaced with an equimolar concentration of *N*-methyl-d-glucamine. Extracellular Ca^2+^ was chelated by addition of EGTA (1 mmol L^−1^) before the cells were stimulated with 0.5 U mL^−1^ thrombin. Results presented are representative of six experiments.

Many studies have exploited the Na^+^ dependence of the NCX to demonstrate that this transporter is present in human platelets (Brass 1984; Rengasamy et al. 1987; Schaeffer and Blaustein 1989). We have previously investigated the role of the NCX in platelet Ca^2+^ signaling by replacing Na^+^ in the extracellular medium with the nonpermeant organic cation, NMDG (Harper and Sage 2007). This manipulation inhibits forward-mode exchange on the NCX, but stimulates reverse-mode exchange by reversing the transmembrane Na^+^ gradient. In the absence of extracellular Ca^2+^, removal of extracellullar Na^+^ should prevent forward-mode exchange and there should be no appreciable reverse-mode exchange. However, replacement of Na^+^ in the extracellular medium will also inhibit other Na^+^-dependent transporters, including the serotonin transporter (SERT) and the Na^+^/H^+^ exchanger (NHE), both of which have previously been suggested to play a role in platelet Ca^2+^ signaling (Siffert and Akkerman 1987; Harper et al. 2009a). Under control conditions, thrombin (0.5 U mL^−1^) evoked the expected rise in SBFI fluorescence (Fig. 2A), previously reported to represent an elevation in [Na^+^]_cyt_ from around 5 mmol L^−1^ to over 27 mmol L^−1^ (Stamouli et al. 1993). Thrombin evoked no change in SBFI fluorescence in the absence of extracellular Na^+^ (0.5 ± 0.4% of control; *n* = 6, *P* < 0.05; Fig. 2A). Replacement of extracellular Na^+^ with NMDG elicited a reduction in thrombin-evoked Ca^2+^ removal into the extracellular medium (68.7 ± 8.4% of control; *n* = 6, *P* < 0.05; Fig. 2B), consistent with inhibition of net forward-mode NCX activity. Na^+^ removal in the absence of extracellular Ca^2+^ replicated the effect of the NCX inhibitors, reducing the initial thrombin-evoked rise in [Ca^2+^]_cyt_ observed over the first min after stimulation (85.9 ± 5.9% of control; *n* = 6, *P* < 0.05; Fig. 2C). However, unlike in the presence of NCX inhibitors, removal of extracellular Na^+^ resulted in the maintenance of a higher [Ca^2+^]_cyt_ for longer than in control cells. Thus, the 3 min integrals of the Ca^2+^ transients were not significantly different between the Na^+^-free and control conditions (114.9 ± 12.9% of control; *n* = 6, *P* > 0.05; Fig. 2C). Similar effects of extracellular Na^+^ removal on thrombin-evoked rises in [Ca^2+^]_cyt_ have been reported by Sanchez et al. (1988). The differences between the thrombin-evoked cytosolic Ca^2+^ signals observed in the presence of NCX inhibitors compared to the absence of extracellular Na^+^ are likely to be due to the inhibition of SERT activity in the latter case. We have previously shown that SERT inhibition significantly prolongs thrombin-evoked cytosolic Ca^2+^ transients (Harper et al. 2009a). These data therefore indicate that the loss of net forward-mode NCX activity elicits a reduction in thrombin-evoked rises in [Ca^2+^]_cyt_ in the absence of extracellular Ca^2+^. We therefore investigated how blocking Ca^2+^ removal from the platelet cytosol results in inhibition, rather than the expected potentiation, of thrombin-evoked Ca^2+^ signals.

### NCX inhibition reduces net Ca^2+^ release from intracellular stores

**Figure 3:**
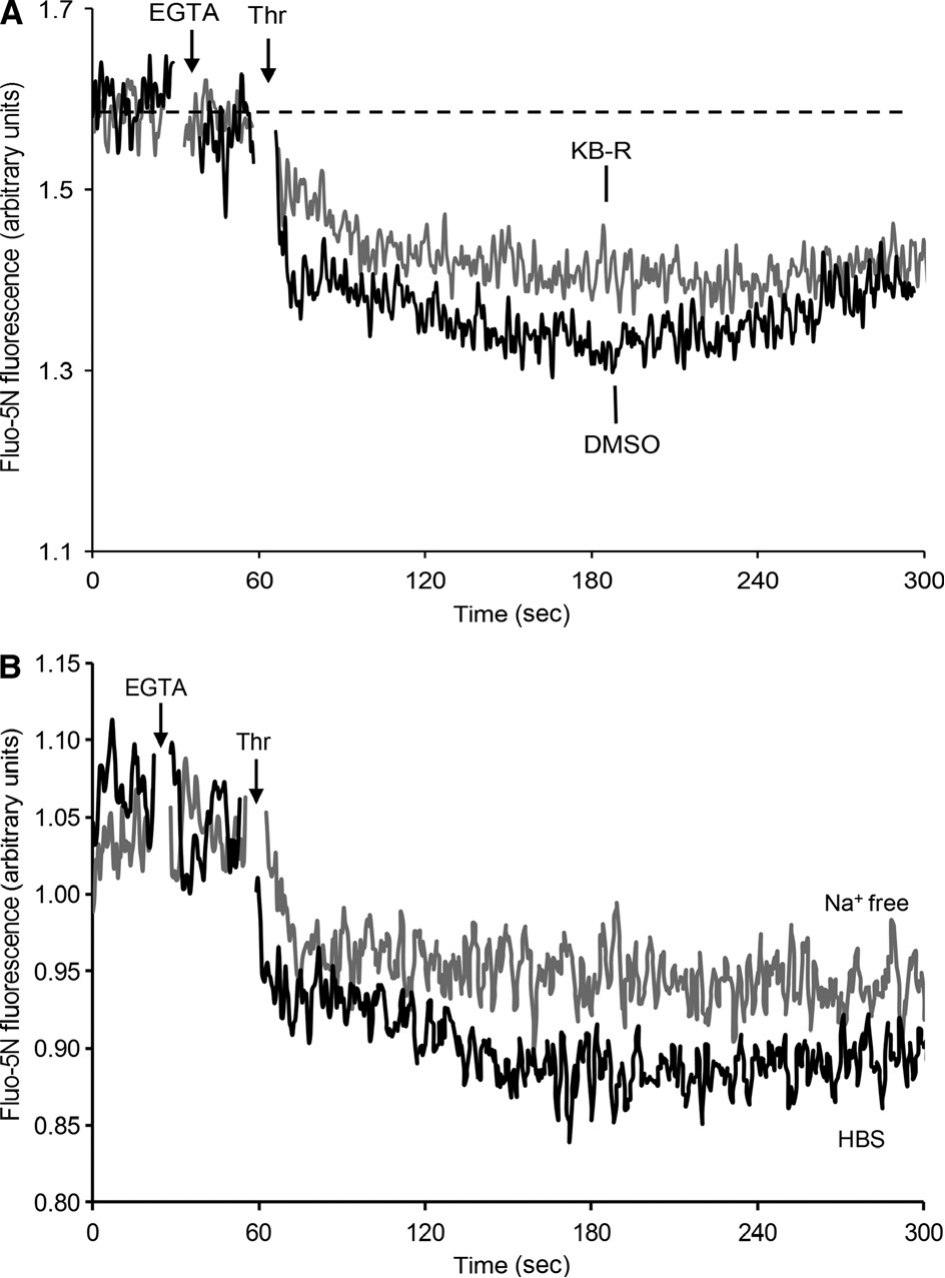
Na^+^/Ca^2+^ exchanger inhibition or replacement of extracellular Na^+^ inhibits thrombin-evoked release of Ca^2+^ from intracellular stores. Fluo-5N-loaded platelets were resuspended in supplemented Hepes-buffered saline (A) or a medium in which extracellular Na^+^ was replaced with an equimolar concentration of *N*-methyl-d-glucamine (B). Extracellular Ca^2+^ was chelated by addition of EGTA (1 mmol L^−1^) before the cells were stimulated with 0.5 U mL^−1^ thrombin. Results presented are representative of six experiments.

The effects of NCX inhibition on thrombin-evoked Ca^2+^ release from intracellular stores was examined in Fluo-5N-loaded cells (Sage et al. 2011). Pretreatment of platelets with 50 μmol L^−1^ KB-R7943 in the absence of extracellular Ca^2+^ slowed and reduced the thrombin-evoked change in [Ca^2+^]_st_ to 63.1 ± 6.2% of control (*n* = 6, *P* < 0.05; Fig. 3A). Replacement of extracellular Na^+^ with NMDG reduced the thrombin-evoked change in [Ca^2+^]_st_ to 68.7 ± 8.4% of control (*n* = 6, *P* < 0.05; Fig. 3B). These results indicate that NCX activity influences thrombin-evoked release of Ca^2+^ from intracellular stores. As Na^+^ removal mimicked the effect of the NCX inhibitor, these data suggest against a nonspecific effect of KB-R7943 on the Ca^2+^ release pathway. This is in agreement with our earlier data, which showed that the inhibitory effect of KB-R7943 on thapsigargin-evoked Ca^2+^ signals could be reversed by restoration of normal autocrine signaling pathways, showing that Ca^2+^ release pathways downstream of these signals were still intact in the presence of the inhibitor (Harper et al. 2009b). These data are discussed further below.

### NCX inhibition slows dense granule secretion

**Figure 4:**
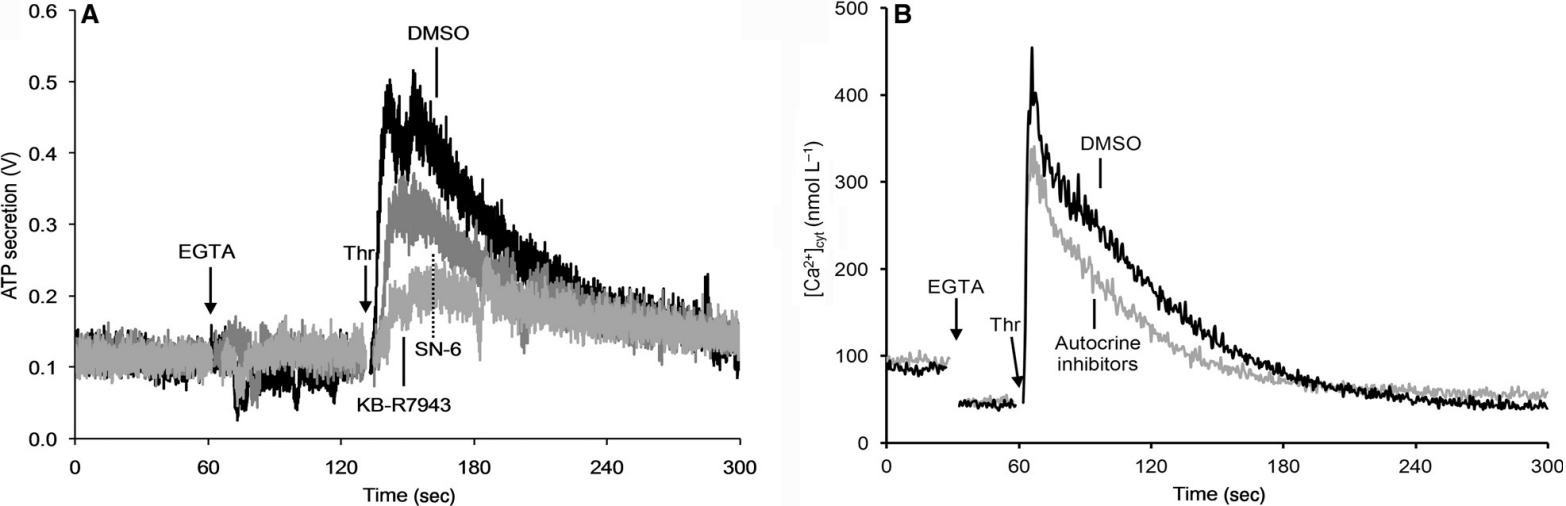
Na^+^/Ca^2+^ exchanger inhibition inhibits thrombin-evoked rises in [Ca^2+^]_cyt_ in part through slowing dense granule secretion. (A) Human platelets suspended in supplemented Hepes-buffered saline (HBS) were preincubated with either 50 μmol L^−1^ KB-R7943, 50 μmol L^−1^ SN-6, or their vehicle, dimethylsulfoxide, for 2 min at 37°C. Extracellular Ca^2+^ was chelated by addition of EGTA (1 mmol L^−1^) before the cells were stimulated with 0.5 U mL^−1^ thrombin. ATP secretion was monitored with luciferin-luciferase. (B) Fura-2-loaded platelets suspended in supplemented HBS were pre-treated with the 5-HT_2A_ antagonist, ketanserin (50 μmol L^−1^), the P_2X1_ antagonist, Ro-0437626 (25 μmol L^−1^), the P_2Y1_ antagonist, 2-methylthioadenosine 5′-monophosphate (100 μmol L^−1^) and the P_2Y12_ antagonist, MRS- 2179 (75 μmol L^−1^), for 2 min at 37°C. Extracellular Ca^2+^ was chelated by addition of EGTA (1 mmol L^−1^) before the cells were stimulated with 0.5 U mL^−1^ thrombin. Data are representative of six experiments.

We have previously demonstrated that the effects of NCX inhibitors on SOCE in human platelets are caused by loss of autocrine activation of the cells due to a slowing of the initial rate of dense granule secretion (Harper et al. 2009b, 2010). Previous work with platelets from patients with Hermansky–Pudlak syndrome, in which dense granules are reduced or absent, has demonstrated a similar deficit in thrombin-evoked Ca^2+^ signaling to that observed in platelets preincubated with NCX inhibitors (Lages and Weiss 1999). Similarly, it has recently been reported that blocking the platelet plasma membrane Ca^2+^-ATPase (PMCA) inhibited agonist-evoked Ca^2+^ signaling as well as dense granule secretion (Jones et al. 2010). Together these results suggest that Ca^2+^ removal across the platelet plasma membrane is required for fast dense granule secretion and thus the full development of agonist-evoked Ca^2+^ signals. Therefore, experiments were conducted to examine the effects of NCX inhibitors on thrombin-evoked ATP release from platelet dense granules using a luciferin–luciferase reporter system. In agreement with our previous studies, pretreatment with either SN-6 (50 μmol L^−1^) or KB-R7943 (50 μmol L^−1^) reduced the extent of dense granule secretion during the first 20 sec after thrombin addition (Fig. 4A). Attempts to examine the effects of Na^+^ removal from the extracellular medium were prevented by the finding that basal luminescence was significantly enhanced in platelet samples incubated in a Na^+^-free medium (109.8 ± 2.4% of control; *n* = 6; *P* < 0.05). The cause of this difference is currently unknown; it could be due to either the loss of Na^+^ or the presence of NMDG in the extracellular medium exerting an effect on either the activity of the luciferin–luciferase reporter or the ATP-scavenging apyrase. Experiments to distinguish these possibilities are needed, but are beyond the scope of this current work. Previous work by other investigators has shown that removal of extracellular Na^+^ inhibits platelet dense granule secretion (Alonso et al. 1989).

### Reduction in autocrine stimulation accounts for a proportion of the observed deficit in thrombin-evoked Ca^2+^ signaling in NCX-inhibited platelets

We have previously reported that the slowing of dense granule secretion, and the resultant reduction in autocrine signaling by the released ADP, ATP, and serotonin, accounts for the effects of NCX inhibitors on SOCE in human platelets (Harper et al. 2009b). Given the deficit in thrombin-evoked Ca^2+^ signaling observed in patients lacking dense granules (Lages and Weiss 1999), and the slowing of thrombin-evoked dense granule secretion caused by NCX inhibition (Fig. 4A), experiments were conducted to examine whether the deficit in thrombin-evoked Ca^2+^ signals observed after NCX inhibition could be accounted for by the loss of dense granule secretion. In agreement with previous studies, simultaneous pretreatment of platelets with the 5-HT_2A_ antagonist ketanserin (50 μmol L^−1^), the P_2X1_ antagonist Ro-0437626 (25 μmol L^−1^), the P_2Y1_ antagonist 2-methylthioadenosine 5′-monophosphate (100 μmol L^−1^), and the P_2Y12_ antagonist MRS-2179 (75 μmol L^−1^) inhibited thrombin-evoked rises in [Ca^2+^]_cyt_ (80.6 ± 2.1% of control; *n* = 6, *P* < 0.05; Fig. 4B). However, this effect was not as pronounced as that observed in the presence of the NCX inhibitors, suggesting that reduced autocrine signaling accounts for some but not all of the observed deficit in thrombin-evoked Ca^2+^ signals following NCX inhibition. Therefore, we considered other mechanisms by which blocking Ca^2+^ removal by the NCX could reduce agonist-evoked Ca^2+^ signals. Among the ideas considered, we hypothesized that Ca^2+^ removed from the platelet cytosol across the plasma membrane is not lost to the bulk medium but is able to accumulate around the cell and recycle back into the cytosol helping to maintain the [Ca^2+^]_cyt_ at higher levels.

### Thrombin evokes an NCX-dependent, sustained rise in pericellular Ca^2+^ concentration

If Ca^2+^ removed from the cell into the extracellular medium can recycle back into the cell, then it should be possible to detect a rise in [Ca^2+^] in the vicinity of the plasma membrane. Thrombin-evoked changes in [Ca^2+^] at the extracellular face of the plasma membrane were examined using the near-membrane Ca^2+^ indicator, FFP-18 (Etter et al. 1996). A similar approach has previously used a different near-membrane indicator, Ca^2+^ green C-18, to measure pericellular Ca^2+^ signals in cardiac myocytes (Blatter and Niggli 1998).

**Figure 5:**
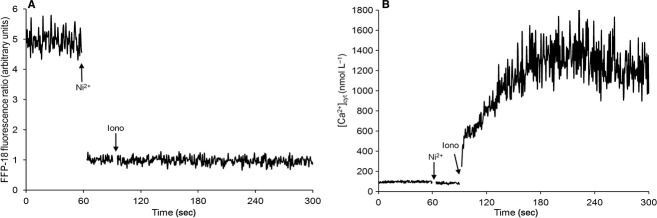
Ionomycin evokes no change in fluorescence in FFP-18-loaded platelets after quenching by extracellular Ni^2+^. FFP-18- (A) or Fura-2-loaded platelets (B) were resuspended in supplemented Hepes-buffered saline. 5 mmol L^−1^ NiCl_2_ was added to quench extracellular indicator before the addition of 1 μmol L^−1^ ionomycin to release Ca^2+^ from intracellular stores.

FFP-18 fluorescence is quenched by the binding of Ni^2+^ (Etter et al. 1996) and the human platelet plasma membrane is impermeable to this divalent cation, even in stimulated cells (Hallam and Rink 1985; Sage and Rink 1987). So, to confirm the extracellular localization of FFP-18, 5 mmol L^−1^ NiCl_2_ was added to rapidly quench any extracellular FFP-18 at the end of each experiment. The resulting signal was compared to the autofluorescence of unloaded cells. We could thus ensure that FFP-18 had not flip-flopped into the inner leaflet of the plasma membrane or entered intracellular compartments. To further exclude the possibility that FFP-18 was exposed to the cytosol, FFP-18-loaded cells were treated with 5 mmol L^−1^ NiCl_2_, followed by 1 μmol L^−1^ ionomycin to release Ca^2+^ from intracellular stores. Ni^2+^ addition resulted in a rapid quench of fluorescence and there was no change in the 340/380 nm fluorescence ratio upon treatment with ionomycin (Fig. 5A). In contrast, with Fura-2-loaded platelets, after 5 mmol L^−1^ Ni^2+^ was used to quench extracellular indicator, ionomycin addition resulted in a rise in the 340/380 nm fluorescence ratio, as expected given the cytosolic location of Fura-2 (Fig. 5B).

**Figure 6:**
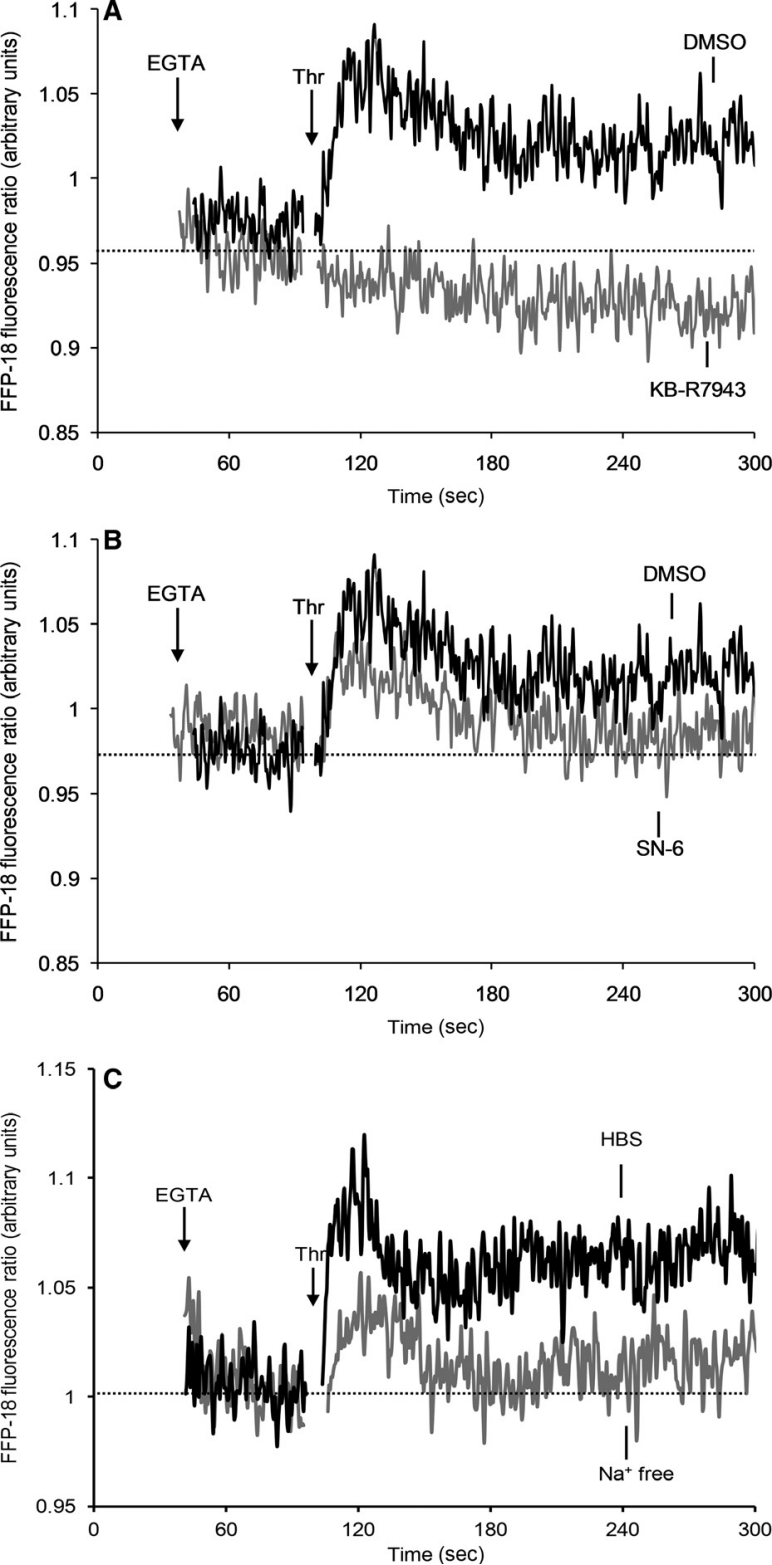
Thrombin evokes an Na^+^/Ca^2+^ exchanger-dependent, sustained rise in pericellular Ca^2+^ concentration. (A, B) FFP-18-loaded human platelets suspended in supplemented Hepes-buffered saline (HBS) were pre-treated with either 50 μmol L^−1^ KB-R7943 (A), 50 μmol L^−1^ SN-6 (B), or their vehicle, dimethylsulfoxide, for 2 min at 37°C. (C) FFP-18-loaded human platelets were suspended in supplemented HBS or a medium in which extracellular Na^+^ was replaced with an equimolar concentration of *N*-methyl-d-glucamine. (A–C) Extracellular Ca^2+^ was chelated by addition of EGTA (1 mmol L^−1^) before the cells were stimulated with 0.5 U mL^−1^ thrombin. Data are representative of 5–9 experiments.

Thrombin stimulation of untreated platelets led to a rapid increase in [Ca^2+^]_peri_ which stayed above basal levels for the duration of the recording (Fig. 6). Thrombin-evoked rises in [Ca^2+^]_peri_ were reduced by preincubation with SN-6 (50 μmol L^−1^; 41.8 ± 6.6% of control; *n* = 5; *P* < 0.05) or removal of extracellular Na^+^ (13.7 ± 10.5% of control; *n* = 9; *P* < 0.05; Fig. 6B and C), while KB-R7943 (50 μmol L^−1^) consistently abolished this response (*n* = 5; Fig. 6A). These data are consistent with the reduction in the thrombin-evoked rise in [Ca^2+^]_cyt_ being due to inhibition of a pericellular Ca^2+^ recycling system.

### Pericellular Ca^2+^ recycling occurs through Ca^2+^-permeable ion channels

**Figure 7:**
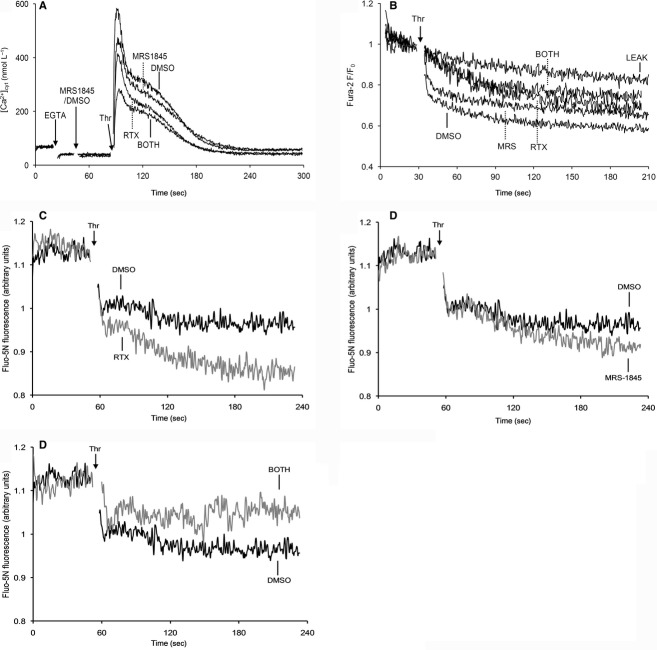
Pericellular Ca^2+^ recycling occurs through Ca^2+^-permeable ion channels. Fura-2- (A, B) or Fluo- 5N- (C–E) loaded platelets were suspended in supplemented Hepes-buffered saline (HBS). (A, C–E) Cells were pre-treated with either 20 μmol L^−1^ 5′-Iodo-RTX or its vehicle, dimethylsulfoxide (DMSO), for 2 min at 37°C. Extracellular Ca^2+^ was chelated by addition of 1 mmol L^−1^ EGTA before addition of either 30 μmol L^−1^ MRS-1845 or its vehicle, DMSO. Platelets were then stimulated 1 min later with 0.5 U mL^−1^ thrombin. Results presented are representative of 6–7 experiments. (B) Fura-2-loaded platelets suspended in a Ca^2+^-free HBS supplemented with 0.1 U mL^−1^ apyrase were preincubated with 20 μmol L^−1^ 5'-Iodo-RTX or its vehicle, DMSO, for 2 min at 37°C. Extracellular Ca^2+^ was chelated by addition of 300 μmol L^−1^ EGTA, followed 30 sec later by 500 μmol L^−1^ MnCl_2_. After another 30 sec, 30 μmol L^−1^ MRS-1845 or its vehicle, DMSO were added. Platelets were stimulated 1 min later with 0.5 U mL^−1^ thrombin.

If pericellular Ca^2+^ recycling occurs then Ca^2+^ must be able to reenter the cell through Ca^2+^-permeable ion channels in the plasma membrane. Therefore, blocking such channels should reduce the thrombin-evoked rise in [Ca^2+^]_cyt_. Indeed, previous work has shown that the broad-spectrum, nonselective cation channel blocker, SKF-96365, and the SOC blocker, econazole, both inhibit thrombin-evoked Ca^2+^ signals elicited in the absence of extracellular Ca^2+^ (Merritt et al. 1990; Alonso et al. 1991). However, most wide spectrum Ca^2+^ channel blockers are known to have secondary effects on other targets such as inositol 1,4,5-trisphosphate (IP_3_) receptors, sarco/endoplasmic reticulum Ca^2+^-ATPases (SERCAs) or other Ca^2+^ removal pathways that complicate the interpretation of the effects of these agents. Therefore, we examined the effects on thrombin-evoked Ca^2+^ signaling of MRS-1845 or 5′-Iodo-resiniferatoxin (5′-Iodo-RTX; Harper et al. 2009a), specific inhibitors of SOCs and TRPV1, respectively. Previous work has shown that blocking P_2X1_ receptors has no effect on thrombin-evoked Ca^2+^ release (Fung et al. 2007), which we confirmed under our conditions using the P_2X1_ inhibitor, Ro-0437626 (data not shown). MRS-1845 (30 μmol L^−1^), 5′-Iodo-RTX (20 μmol L^−1^) or both in combination reduced thrombin-evoked rises in [Ca^2+^]_cyt_ in the absence of extracellular Ca^2+^ to 76.2 ± 6.0%, 70.1 ± 5.0% or 54.4 ± 3.0% of control, respectively (all *P* < 0.05, *n* = 7; Fig. 7A). Thrombin-evoked Mn^2+^ entry was also inhibited by these compounds (83.3 ± 4.0%, 63.3 ± 6.5% or 51.5 ± 6.2% of control for MRS-1845, 5′-Iodo-RTX and both compounds, respectively; all *P* < 0.05, *n* = 6; Fig. 7B), in line with their ability to block Ca^2+^-permeable ion channels in the platelet plasma membrane. These compounds blocked thrombin-evoked rises in [Ca^2+^]_cyt_ in proportion to their effects on thrombin-evoked Mn^2+^ entry. Neither inhibitor alone reduced the thrombin-evoked fall in [Ca^2+^]_st_ as measured in Fluo-5N-loaded cells (99.4 ± 7.6% or 125.8 ± 9.8% of control for MRS-1845 or 5′-Iodo-RTX, respectively; *P* < 0.05 for 5′-Iodo-RTX, *n* = 6; Fig. 7C and D), thus suggesting neither compound directly inhibits IP_3_-mediated Ca^2+^ release and that their effects must be related to their ability to prevent Ca^2+^ entering the cell. As pericellular Ca^2+^ is the only source of extracellular Ca^2+^ available to enter the cells under these experimental conditions, these results further suggest that Ca^2+^ that is removed across the plasma membrane is able to recycle back into the cell through Ca^2+^-permeable ion channels such as TRPV1 and SOCs. However, when both inhibitors were added simultaneously, the thrombin-evoked fall in [Ca^2+^]_st_ was inhibited (76.4 ± 7.6% of control; *n* = 6; *P* < 0.05; Fig. 7E) to a similar extent to that seen with NCX inhibitors previously. These results suggest that interference with Ca^2+^ recycling has a complex effect on intracellular Ca^2+^ store dynamics, with small reductions in recycling reducing store refilling via the smaller rise in [Ca^2+^]_cyt_, whereas larger reductions in Ca^2+^ recycling cause a reduction in Ca^2+^ release, either through a reduction in a direct effect of Ca^2+^ on the IP_3_ receptor or an indirect effect via changes in granule secretion, phosphoinositide metabolism or a change in Ca^2+^ reuptake elicited by a Ca^2+^-dependent effector such as protein kinase C. These possibilities are discussed further in relation to the quantitative analysis below.

### Interference with the pericellular Ca^2+^ signal reduces thrombin-evoked rises in [Ca^2+^]_cyt_

**Figure 8:**
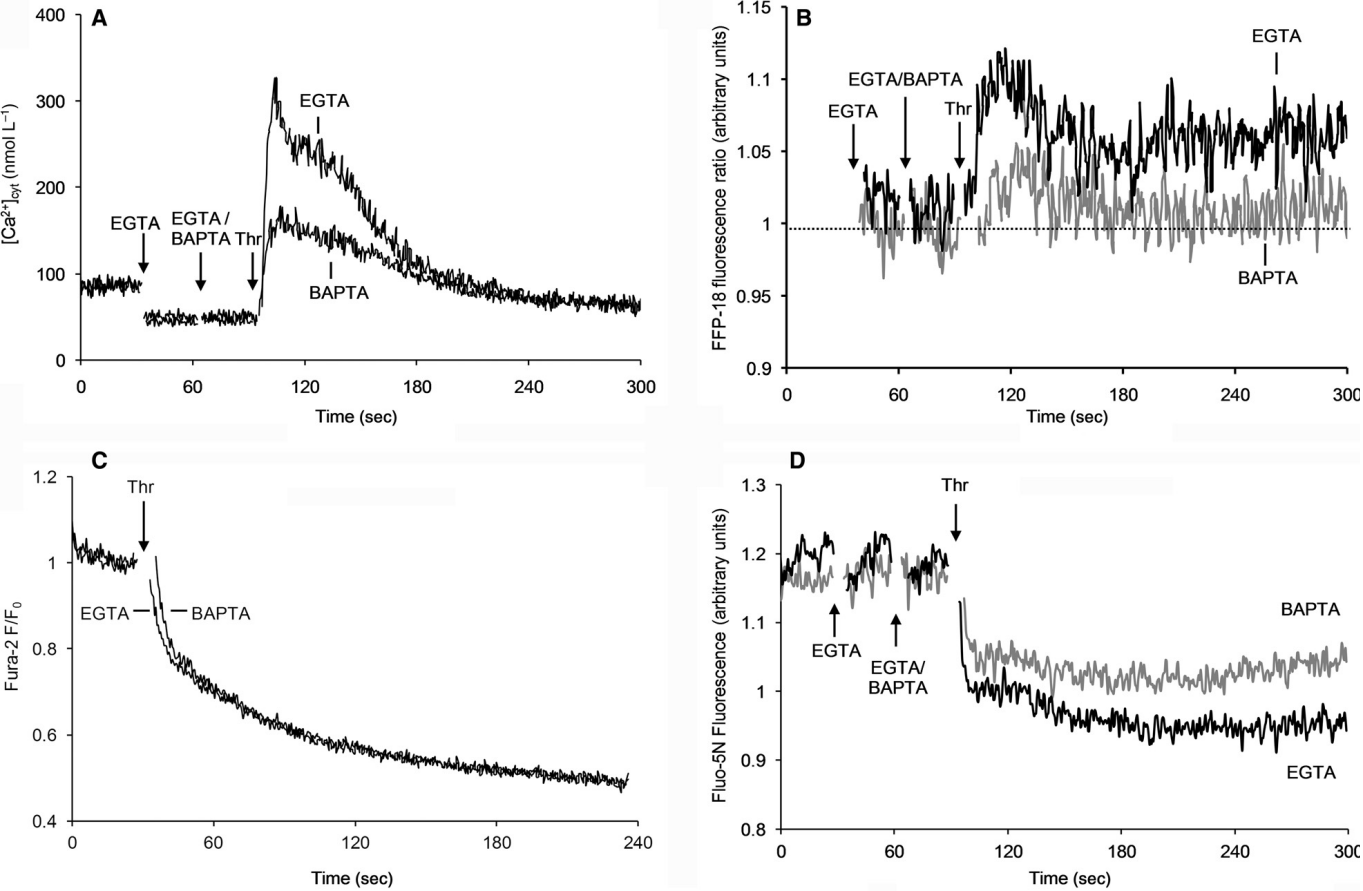
Buffering the thrombin-evoked pericellular Ca^2+^ rise with fast Ca^2+^ chelators inhibits thrombin-evoked rises in [Ca^2+^]_cyt_. Fura-2- (A), FFP-18- (B) or Fluo-5N- (D) loaded human platelets were suspended in supplemented Hepes-buffered saline (HBS). Extracellular Ca^2+^ was chelated by the addition of 1 mmol L^−1^ EGTA. The fast Ca^2+^ chelator, BAPTA (1 mmol L^−1^), or an equal additional concentration of the slower chelator EGTA were then added before the cells were stimulated with 0.5 U mL^−1^ thrombin. (C) Fura-2-loaded cells were suspended in a Ca^2+^-free HBS supplemented with 0.1 U mL^−1^ apyrase. Extracellular Ca^2+^ was chelated by addition of 300 μmol L^−1^ EGTA and 30 sec later either 1 mmol L^−1^ BAPTA or a further 1 mmol L^−1^ EGTA was added. 30 sec later 500 μmol L^−1^ MnCl_2_ was added and then, after another 30 sec, the platelets were stimulated with 0.5 U mL^−1^ thrombin.

To further test whether a pericellular Ca^2+^ recycling system can explain the effects of NCX inhibition on the thrombin-evoked Ca^2+^ signal, experiments were performed attempting to interfere with the generation of the pericellular Ca^2+^ signal. Two different experimental approaches were used. The first compared the effects of adding equimolar concentrations of the fast-onset Ca^2+^ chelators, BAPTA or dimethyl-BAPTA, in place of the slow-onset Ca^2+^ chelator EGTA. The faster speed of buffering allows the BAPTA series chelators to buffer Ca^2+^ more effectively in the vicinity of the source of the Ca^2+^ flux. The difference in Ca^2+^ binding kinetics should allow BAPTA chelators to be significantly more effective than EGTA in buffering Ca^2+^ in the pericellular region of the cell (as previously discussed by Deisseroth et al. (1996) utilizing material from Stern (1992)). When cells were preincubated with 1 mmol L^−1^ BAPTA in place of equimolar EGTA, thrombin-evoked rises in both [Ca^2+^]_cyt_ (72.8 ± 1.9% of control; *n* = 6; *P* < 0.05; Fig. 8A) and [Ca^2+^]_peri_ were significantly reduced (54.5 ± 9.8% of control; *n* = 7, *P* < 0.05, Fig. 8B). A similar deficit in the thrombin-evoked rise in [Ca^2+^]_cyt_ was observed when cells were preincubated with 100 μmol L^−1^ dimethyl-BAPTA (62.8 ± 5.1% of control; *n* = 6, *P* < 0.05; data not shown). As the K^+^ salts of the chelators are cell impermeant, the effects on pericellular Ca^2+^ must cause the effects on [Ca^2+^]_cyt_. BAPTA chelators have been shown to have nonspecific effects on K^+^ channels when loaded intracellularly (Urbano and Buño 1998; Tang et al. 2007). Blockade of K^+^ channels would be expected to depolarize the platelet membrane potential (Mahaut-Smith et al. 1990). To rule out any such nonspecific actions of BAPTA application, experiments were performed to examine the relative effects of EGTA and BAPTA on the thrombin-evoked increase in Mn^2+^ entry into Fura-2-loaded platelets. Any membrane depolarization would be expected to reduce Mn^2+^ entry. As shown in Figure 8C, incubation with BAPTA had no significant effect on thrombin-evoked Mn^2+^ entry into the cells when compared to incubation with an equimolar concentration of EGTA (93.3 ± 6.0% of control; *n* = 6, *P* > 0.05). These data therefore suggest that intracellular signal transduction leading to Ca^2+^ permeable ion channel activation by thrombin is normal in the presence of BAPTA, but extracellular interference with the Ca^2+^ flux leads to a reduction in the rise in [Ca^2+^]_cyt_.

Experiments were also performed to investigate whether faster buffering of the pericellular Ca^2+^ signal affects the thrombin-evoked fall in [Ca^2+^]_st_ in an analogous manner to NCX inhibition or blockage of Ca^2+^-permeable ion channels in the plasma membrane (Figs. 3, 7E). Addition of BAPTA to the extracellular medium before stimulation of platelets with thrombin significantly inhibited the thrombin-evoked fall in [Ca^2+^]_st_ in comparison with cells pretreated with the same concentration of EGTA (75.0 ± 6.8% of control; *n* = 6; *P* < 0.05; Fig. 8D). These data therefore confirm that a reduction in pericellular Ca^2+^ recycling is able to reduce agonist-evoked Ca^2+^ release, presumably through an effect on a Ca^2+^-dependent feedback pathway which amplifies the initial release.

**Figure 9:**
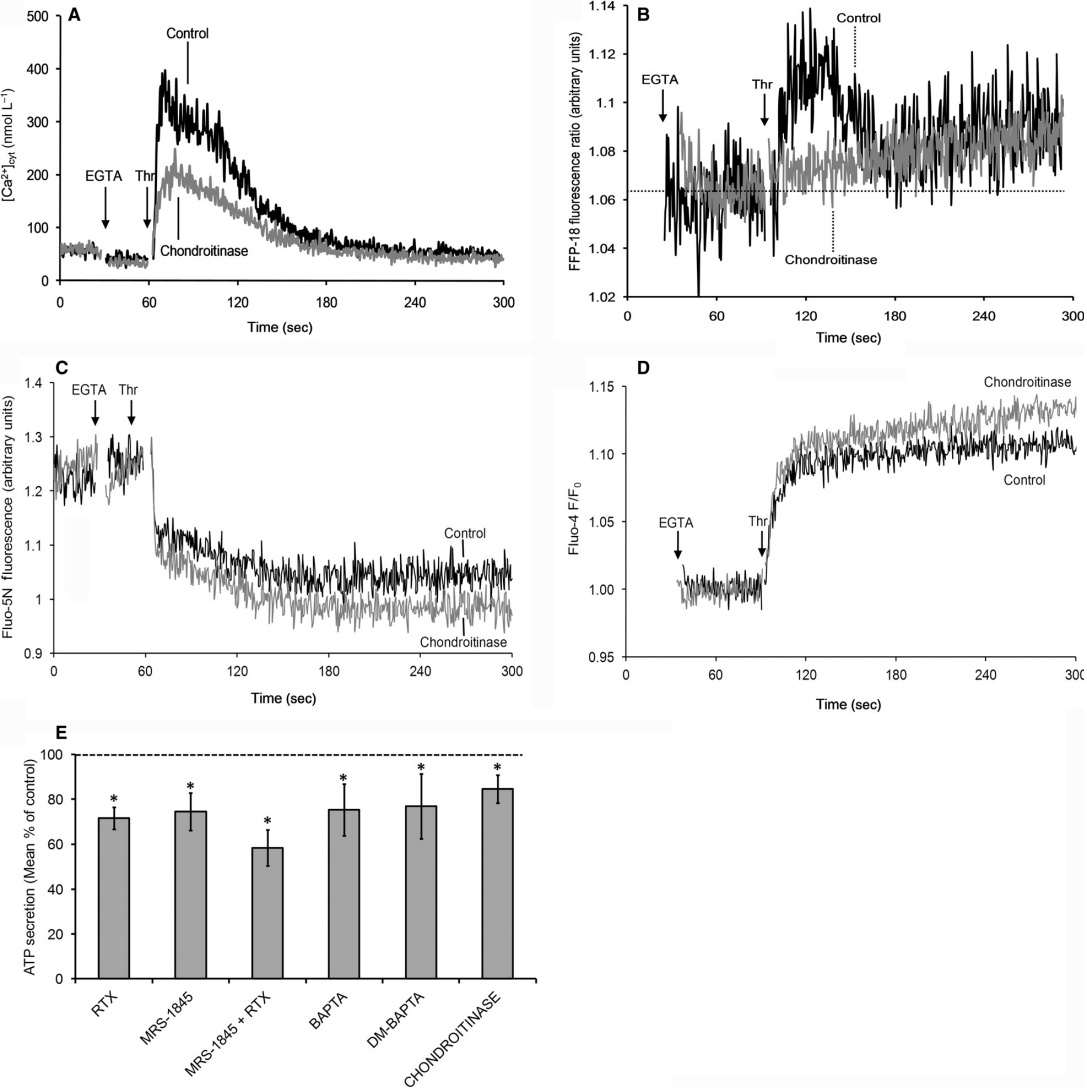
Degradation of the platelet glycocalyx by pre-treatment with chondroitinase inhibits thrombin-evoked rises in [Ca^2+^]_cyt_ by interfering with thrombin-evoked pericellular Ca^2+^ accumulation. Fura-2- (A), FFP-18- (B) or Fluo-5N- (C) loaded human platelets were treated with 1 U mL^−1^ chondroitinase ABC lyase or its vehicle, Hepes-buffered saline (HBS), for 1 h as previously described (Steiner 1986). (A, B, C) Platelets were resuspended in supplemented HBS and extracellular Ca^2+^ was chelated by addition of 1 mmol L^−1^ EGTA before the cells were stimulated with 0.5 U mL^−1^ thrombin. (D) Platelets treated with 1 U mL^−1^ chondroitinase ABC lyase or its vehicle, HBS, for 1 h were suspended in supplemented HBS with 2.5 μmol L^−1^ Fluo-3. Extracellular Ca^2+^ was chelated by addition of 1 mmol L^−1^ EGTA and the platelets were stimulated 1 min later with 0.5 U mL^−1^ thrombin. Results presented are representative of 6–9 experiments. (E) ATP secretion from platelets treated as indicated and described in the legends to Figures 7 and 8, was monitored with luciferin-luciferase. Results are means ± SEM of six measurements. *P < 0.05.

Previous work by Steiner (1986) demonstrated that degrading the platelet glycocalyx by incubating cells with chondroitinase significantly reduced platelet cytosolic Ca^2+^ signals elicited in the absence of extracellular Ca^2+^. These results were suggested to be due to the ability of the negatively charged glycocalyx to store Ca^2+^ in the close vicinity of the plasma membrane. Therefore, experiments were performed to examine whether the effect of chondroitinase pretreatment on cytosolic Ca^2+^ signals could be due to inhibition of thrombin-evoked rises in [Ca^2+^]_peri_. Chondroitinase pretreatment significantly inhibited thrombin-evoked rises in both [Ca^2+^]_cyt_ (72.2 ± 4.0% of control; *n* = 9, *P* < 0.05; Fig. 9A) and [Ca^2+^]_peri_ (53.7 ± 7.9% of control; *n* = 7, *P* < 0.05; Fig. 9B). The action of chondroitinase appeared to be specific to the thrombin-evoked rise in [Ca^2+^]_peri_ rather than an effect on thrombin-evoked signal transduction pathways, as the thrombin-evoked fall in [Ca^2+^]_st_ (Fig. 9C) and the rise in [Ca^2+^]_ext_ (Fig. 9D) were both potentiated by chondroitinase treatment (133.9 ± 12.7% and 128.7 ± 10.9% of control, respectively; both *n* = 6, *P* < 0.05) These results indicate that although Ca^2+^ release from the intracellular stores was not inhibited by chondroitinase treatment, Ca^2+^ that was removed from the cells, instead of staying close to the plasma membrane, rapidly diffused away into the bulk extracellular medium, as indicated by the greater rise in [Ca^2+^]_ext_ (Fig. 9D), thus preventing its reentry back into the cell. This could explain the reduced [Ca^2+^]_cyt_ and, by reducing Ca^2+^ available for store refilling, the greater decrease in [Ca^2+^]_st_ after chondroitinase treatment.

### Interrupting pericellular Ca^2+^ recycling slows dense granule secretion

Experiments were next conducted to investigate whether pericellular Ca^2+^ recycling influences dense granule secretion. The effect on ATP secretion from dense granules was investigated in platelets preincubated with fast Ca^2+^ chelators, chondroitinase or blockers of Ca^2+^-permeable ion channels. Blockade of any of the components of the Ca^2+^ recycling system reduced the extent of dense granule secretion during the first 20 sec after thrombin addition (Fig. 9E), supporting the idea that pericellular Ca^2+^ recycling across the plasma membrane plays a role in accelerating dense granule secretion. These data provide a possible explanation for the role of the NCX in platelet dense granule secretion (Harper et al. 2009a), through its ability to create a pericellular Ca^2+^ signal.

### Single cell imaging of thrombin-evoked changes in extracellular Fluo-4 fluorescence confirms the presence of thrombin-evoked pericellular Ca^2+^ signals

**Figure 10:**
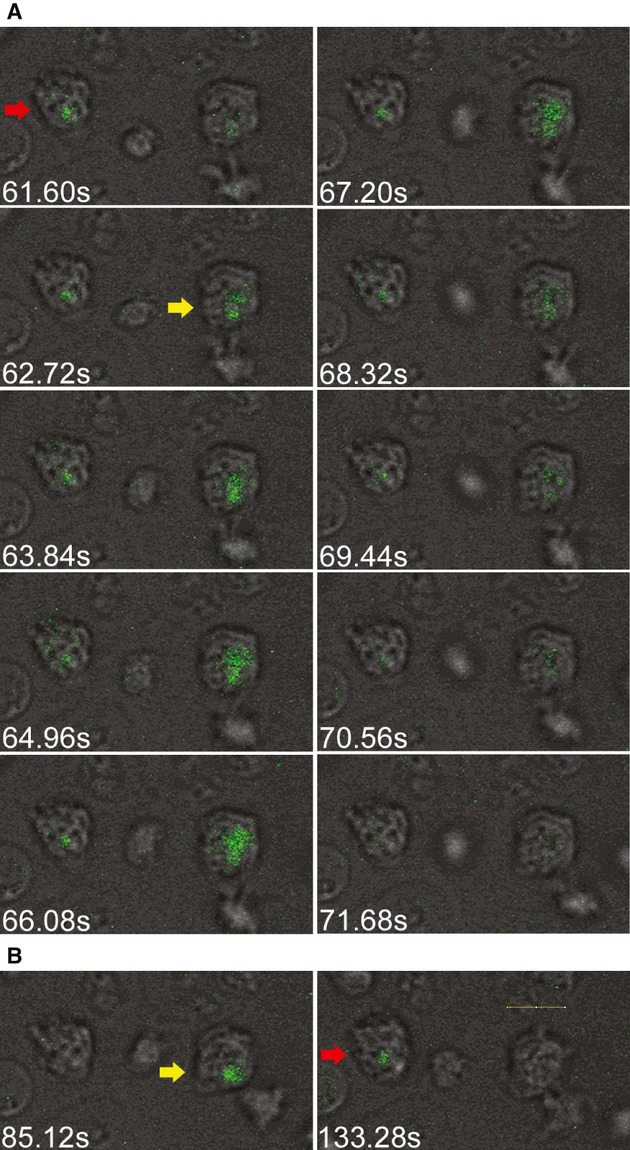
Single cell imaging of extracellular Ca^2+^ signals. Washed human platelets in Ca^2+^-free Tyrodes solution (1 × 10^8^ cells mL^−1^) were pipetted into chambered coverslips coated with fibrinogen and allowed to adhere for 15 min for observation by confocal microscopy. 2.5 μmol L^−1^ Fluo-4 was then added and the platelets stimulated with 0.5 U mL^−1^ thrombin. (A) shows successive frames recorded 61.60–71.68 sec into the observation period. Cells showing representative features are indicated with coloured arrows at the point they begin to show extracellular Ca^2+^ signals. The red arrow indicates a cell in which the extracellular Ca^2+^ signal remained spatially static, being maintained in the same area over a period of time. The yellow arrow indicates a cell in which the initial microdomain of extracellular Ca^2+^ spread in a restricted manner down just one half of the cell. (B) shows frames recorded later where extracellular Ca^2+^ signals were generated in the same subdomains that were observed to generate signals earlier in the experiment (A), as indicated by the yellow or red arrows. The yellow scale bar in the 133.28 sec panel represents represents 3.5 μm.

To further elucidate the presence and location of the pericellular Ca^2+^ signal, confocal microscopy was used to examine the Ca^2+^ concentration in the extracellular medium around single, washed human platelets exposed to the cell-impermeant Ca^2+^ indicator, Fluo-4, in the absence of extracellular Ca^2+^. Platelets allowed to settle on fibrinogen-coated slides did not show pericellular Ca^2+^ signals (not shown). Addition of thrombin evoked pericellular Ca^2+^ signals at some point during the 5 min of observation (Fig. 10). Pericellular Ca^2+^ signals similar to those evoked by thrombin were also observed in cells adhering to CRP (data not shown).

Agonist-evoked cytosolic Ca^2+^ signals in fibrinogen-bound platelets are reported to rise almost instantaneously throughout the entire cell (Heemskerk et al. 2001). In contrast, pericellular Ca^2+^ signals were associated with specific microdomains of the cell, in an eccentric location within the cell body, and did not always spread from these points. In some adherent platelets, the initial region of increased extracellular [Ca^2+^] spread within the observed boundaries of the cells. However, in contrast to cytosolic Ca^2+^ signals (Heemskerk et al. 2001), the spread of pericellular Ca^2+^ could be seen in some cells to be in restricted directions and generally did not encompass the entire cell. Furthermore, the pericellular Ca^2+^ signal spread across the cell more slowly than cytosolic Ca^2+^ signals, taking about 4.5 sec to go from one side to the other in the example indicated by the yellow arrow in Figure 10A, compared to the almost instantaneous spread of Ca^2+^ through the platelet cytosol (Heemskerk et al. 2001). Interestingly, agonist-evoked pericellular Ca^2+^ signals only infrequently spread beyond the observed boundary of the platelet plasma membrane as indicated by overlay of Fluo-4 fluorescence and transmitted light images (Fig. 10). A quantitative analysis of the location of the thrombin-stimulated Fluo-4 fluorescence signals was conducted on 21 cells. The mean fluorescence intensity generated in areas within the platelet boundary was compared against the mean fluorescence observed in both the pericellular region surrounding the cell and the mean background fluorescence signal observed from areas without any nearby cell. This analysis showed that the mean Fluo-4 fluorescence rise of pixels inside the platelet boundary was 5.22 ± 0.64 units, which was significantly greater than both the rise observed in the pericellular region around these cells (0.35 ± 0.03 units; *P* < 0.05) and the background fluorescence (0.28 ± 0.02 units; *P* < 0.05). In contrast, the mean background fluorescence was not significantly different from that observed in the pericellular region around the stimulated cells (*P* < 0.05). Therefore, the pericellular Ca^2+^ signals appeared to be generated in a restricted volume within the platelet which was contiguous with the extracellular medium. Platelets contain a tubular invagination of their plasma membrane, the open canalicular system (OCS), which resembles the transverse-tubule system of striated muscle types (White 1972). The OCS would provide an appropriate location for the generation of the observed pericellular Ca^2+^ signals as Ca^2+^ accumulated within this system would appear to be localized within the interior of the platelet and in a microdomain due to the small diameter of the tubules of the OCS. Furthermore, the tubules of the OCS would constrain the speed and direction of Ca^2+^ diffusion as observed here. Our data therefore suggest that Ca^2+^ removal from the platelet is principally localized to the membrane of the open canalicular system and that removal across the true surface membrane has a limited role in platelet Ca^2+^ dynamics. This is consistent with previous data from Cutler et al. (1980), who demonstrated that Ca^2+^-ATPase activity is limited to the membranes of the open canalicular and dense tubular systems, in contrast to the Na^+^/K^+^ ATPase, which is predominantly found at the true surface membrane. An alternative explanation for the generation of a pericellular Ca^2+^ signal would be that Ca^2+^ was removed across the upper or lower surface of the adherent platelets. Under these conditions, however, it would be expected that Ca^2+^ would be free to diffuse relatively rapidly in all directions and would be unlikely to stay localized within a microdomain.

In cells that were observed to have more than one rise in [Ca^2+^]_peri_ during the period of observation, the pericellular Ca^2+^ signals reappeared in the same subregions of the platelet (compare images at 61.60 sec and 62.72 sec in Figure 10A to 133.28 sec and 85.12 sec in Figure 10B for the cells indicated by the red and yellow arrows, respectively). These observations suggest the possibility that a specific region of the cell is involved in creating the microdomains of elevated pericellular Ca^2+^. The cellular basis underlying the creation of these microdomains is considered further in the discussion.

### Single cell imaging of FFP-18 fluorescence

**Figure 11:**
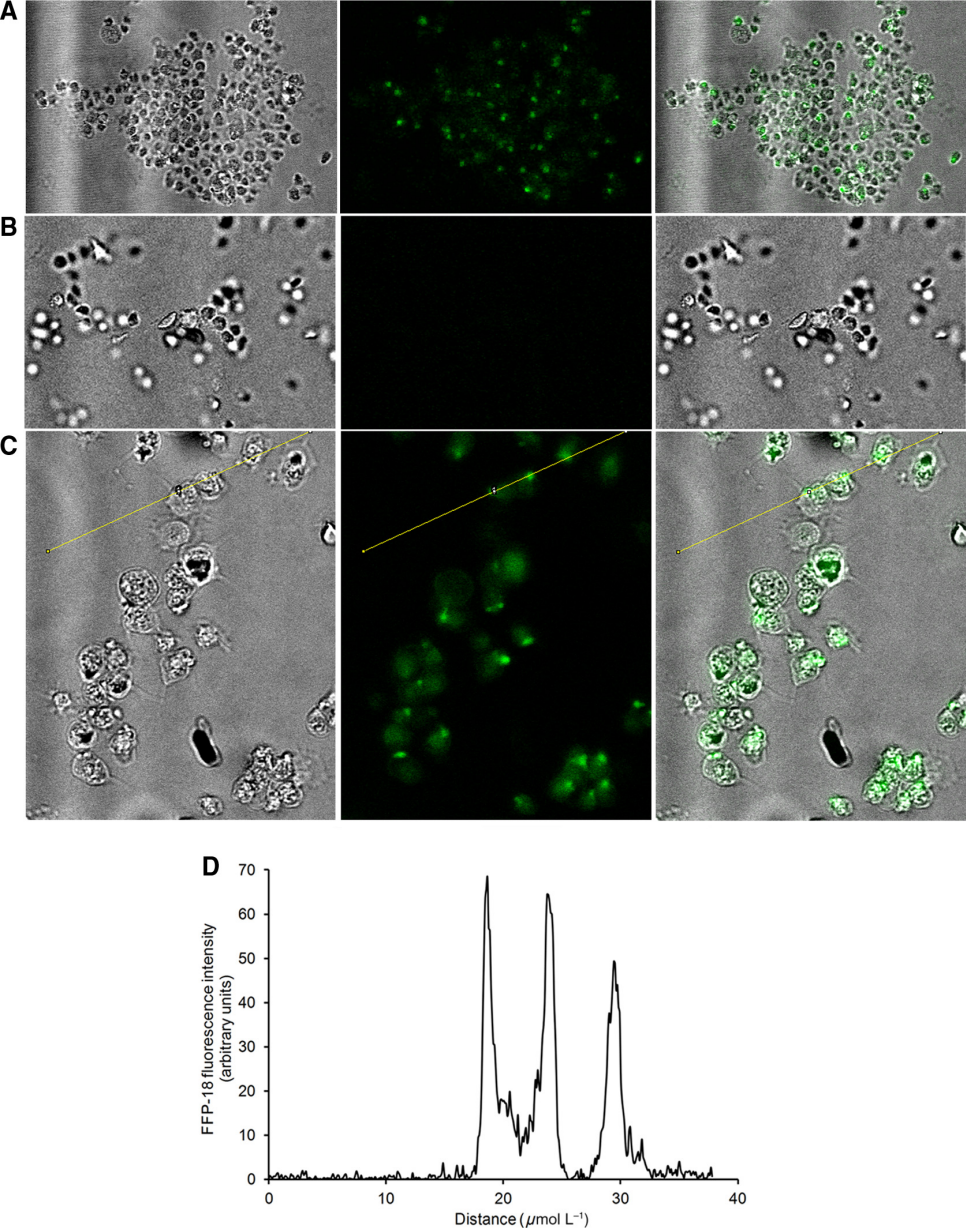
Single cell imaging of FFP-18-loaded platelets. FFP-18-loaded platelets resuspended in Hepes-buffered saline with 1 mmol L^−1^ EGTA were allowed to adhere to poly-l-lysine coated coverslips before another coverslip was placed on top to permit the use of a water immersion lens without disturbing the cells. FFP-18 fluorescence was monitored using a Leica SP-5 confocal microscope with an excitation wavelength of 405 nm and emission wavelengths of 420–580 nm. Left-hand panels show transmitted light images, centre panels fluorescence images and right-hand panels overlays of these two. (A) and (C) show images from FFP-18-loaded platelets at low and high magnification. (B) shows images from FFP-18-loaded platelets after the addition of 5 mmol L^−1^ NiCl2. (D) shows the measured FFP-18 fluorescence observed across the yellow line indicated in (C).

Experiments were performed to examine the distribution of fluorescence in FFP-18 loaded cells to see whether this dye loads solely into the platelet surface membrane or whether it also had access to the extracellular face of the OCS. FFP-18-loaded platelets suspended in supplemented HBS containing 1 mmol L^−1^ EGTA were allowed to adhere to poly-l-lysine-coated coverslips for imaging. FFP-18 fluorescence was imaged using an excitation wavelength of 405 nm. At this wavelength, fluorescence is enhanced by low [Ca^2+^], giving the best sensitivity to examine the distribution of the dye. These images showed that fluorescence was not solely limited to the platelet surface membrane but could also be visualized as a high intensity, eccentrically placed hotspot in each cell, as well as being diffusely distributed at lower intensity in the surface membrane and throughout the rest of the platelet (Fig. 11A and C). The diffuse nature of the FFP-18 fluorescence was confirmed by examining a variety of linescans through FFP-18-loaded cells (Fig. 11D; linescan marked by yellow line in Fig. 11C). Fluorescence increased sharply when the scan crossed an area containing a loaded platelet, as compared to the nearby background signal, and there was no clear distinction in the fluorescence between closely adjacent platelets toward the center of the scan, suggesting that fluorescence spreads right through the cells. Imaging of unloaded cells found no sign of any autofluorescence at the photomultiplier tube voltages (around 15% of maximum) used to examine the FFP-loaded cells, suggesting that the fluorescence observed comes specifically from the FFP-18. Addition of 5 mmol L^−1^ Ni^2+^ to the platelets prior to imaging fully quenched all observable fluorescence (Fig. 11B). This result supports the data presented earlier in Figure 5, which show that FFP-18 loads exclusively into the extracellular leaflet of the plasma membrane.

For imaging experiments, platelets were attached to poly-l-lysine-coated coverslips, resulting in some activation and spreading of the cells. The spread cells have limited thickness, making high-resolution 3D reconstructions difficult. We have obtained *Z*-stacks which have been assembled into 3D images. These show regions of intense FFP-18 fluorescence which pass through the cells, compatible with the presence of FFP-18 in the membrane of the OCS, as well as in the surface membrane (see Video S1). These data are consistent with FFP-18 loading into the extracellular face of both the surface and invaginated OCS membranes. The diffuse low-intensity fluorescence of the FFP-18 indicator is consistent with the OCS spreading from one side to the other of the platelet, as previously demonstrated in 3D electron tomographic reconstructions of this invaginated membrane system (van Nispen tot Pannerden et al. 2010). The eccentrically positioned hotspots of high-intensity FFP-18 fluorescence (Fig. 11C) would be compatible with the reported accumulation of OCS channels at membrane complexes, formed by the close apposition of the platelet dense tubular system (DTS; the platelet equivalent of the endoplasmic reticulum) and the OCS at eccentric locations in the platelet interior (White 1972; van Nispen tot Pannerden et al. 2010).

The hotspots in the FFP-18 images were found to contain 23.2% ± 1.6% of the total cellular FFP-18 fluorescence (*n* = 18) and measured 758 ± 39 nm in diameter (*n* = 17). Interestingly, the microdomains of high [Ca^2+^]_peri_ in stimulated cells were found to be of an equivalent size (710 ± 32 nm; *n* = 29). The agonist-evoked pericellular Ca^2+^ signals observed in the extracellular Fluo-4 experiments and the hotspots observed in the FFP-18 images were both observed in eccentric microdomains of the cells. One might conjecture that the cellular structure associated with the FFP-18 accumulation is likely to be responsible for the generation of the pericellular Ca^2+^ microdomain. As the DTS is the site of IP_3_-mediated Ca^2+^ release in platelets, the membrane complexes formed by the association of the OCS and DTS would provide a suitable cellular structure for the efficient creation of the pericellular Ca^2+^ signals observed in our experiments.

### Quantitative analysis of the plausibility of a pericellular calcium recycling system

Finally, we quantitatively examined the feasibility of a pericellular Ca^2+^ recycling system in human platelets. To do this, we obtained estimates of the Ca^2+^ binding capacity of the platelet intracellular stores and the platelet cytosol and then used these values to quantitatively assess calibrated measurements of thrombin-evoked rises in [Ca^2+^]_cyt,_ [Ca^2+^]_peri_, [Ca^2+^]_ext,_ and [Ca^2+^]_st_ made from cells from two individual donors.

The calibrated data sets presented below measure the free Ca^2+^ concentrations in the intracellular stores and cytosol and examine the agonist-evoked changes in the *free* Ca^2+^ concentration in these compartments. In contrast, the measurements of extracellular Ca^2+^ accumulation are calibrated to determine the increase in *total* Ca^2+^ concentration (that both in free form as well as that bound to extracellular buffers) as Ca^2+^ leaves the cells. Any change in free [Ca^2+^] in any compartment inside the cell represents a small proportion of the Ca^2+^ moving into or out of that compartment, due to the ability of proteins and membranes to bind Ca^2+^. To estimate the total Ca^2+^ passing into or out of a given cellular compartment requires an estimate of the Ca^2+^-binding capacity of that compartment. We therefore used a minor modification in the methodology of Mogami et al. (1999) to estimate the Ca^2+^ binding capacity of both the platelet cytosol and the intracellular Ca^2+^ stores.

Platelets coloaded with Fluo-5N and either Fura-2, Fura-4F or Fura-FF were used to make measurements of [Ca^2+^]_st_ and [Ca^2+^]_cyt_, respectively, from 1.5-mL aliquots of platelet suspensions (cell density = 3 × 10^8^ cells mL^−1^). In addition, [Ca^2+^]_ext_ was measured in some experiments by addition of 2.5 μmol·mL^−1^ Fluo-4 K^+^ salt to the extracellular medium. In these experiments, there was no interference in the Fluo-4 signal from the Fluo-5N due to the extracellular dye being monitored at a significantly lower photomultiplier tube voltage, which means that changes in Fluo-5N signal were not detectable under these conditions.

### Estimation of calcium-binding capacity of the platelet intracellular calcium stores

**Figure 12:**
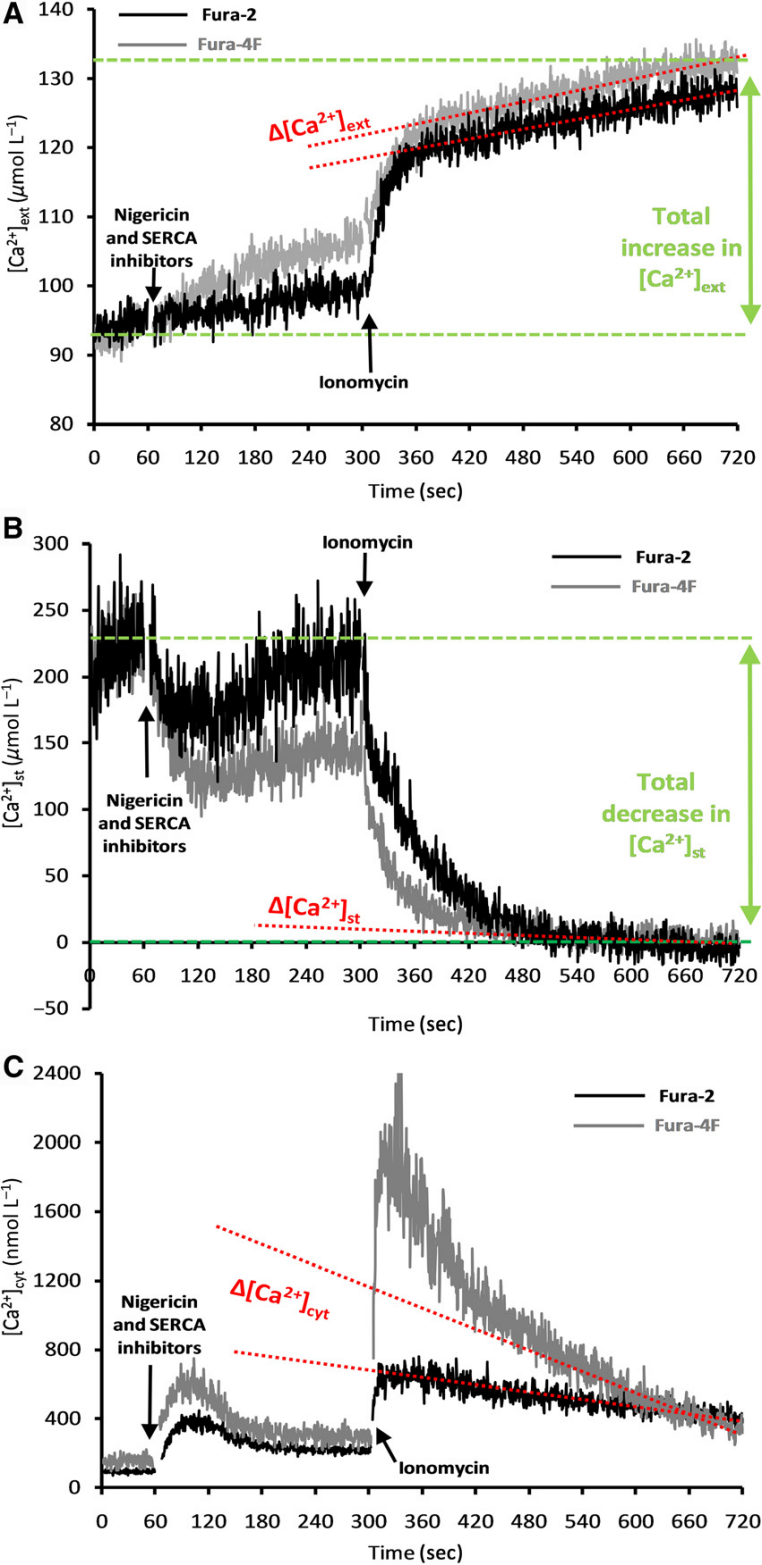
Estimation of Ca^2+^-binding capacity of the platelet cytosol and intracellular Ca^2+^ stores. Platelet Rich Plasma was prepared from freshly donated human blood. The cells were loaded with Fluo-5N by incubation with 250 nmol L^−1^ Fluo-5N/AM for 90 min at 37°C, and subsequently loaded with either Fura-2, Fura-4F or Fura-FF (data not shown) by incubation with 2 μmol L^−1^ of the respective dye for a further 30 min, or incubated with an equivalent volume of the vehicle, dimethylsulfoxide (data not shown). Cells were collected by centrifugation and resuspended in Ca^2+^-free Hepes-buffered saline, to which 2.5 μmol L^−1^ Fluo-4 K^+^ salt was added when required. The cells were stimulated with 10 μmol L^−1^ nigericin, 1 μmol L^−1^ thapsigargin, and 20 μmol L^−1^ TBHQ for 5 min to block Ca^2+^ reuptake into the intracellular Ca^2+^ stores, then 1 μmol L^−1^ ionomycin was added to fully empty the intracellular stores.

Using the above approach, Ca^2+^ fluxes were monitored across each of the platelet cellular compartments in response to artificial depletion of the intracellular Ca^2+^ stores by treating the cells with 10 μmol L^−1^ nigericin, 1 μmol L^−1^ thapsigargin, 20 μmol L^−1^ 2,5 di-(tertbutyl)-1,4-hydroquinone (TBHQ), and 1 μmol L^−1^ ionomycin in Ca^2+^-free medium (Fig. 12).

This method for inducing Ca^2+^ signals prevents complication from significant Ca^2+^ entry from the extracellular medium or sequestration back into the intracellular stores, thus Ca^2+^ flux should be unidirectional from the intracellular stores through the cytosol to the extracellular medium, allowing estimation of the total amount of Ca^2+^ contained within the intracellular stores of the platelet (Fig. 12A). This information, combined with measurement of the total fall in the concentration of Ca^2+^ in the intracellular stores ([Ca^2+^]_st_; Fig. 12B) and previously published data concerning the volume of the platelet dense tubular system, dense granules, and alpha granules (Fromjovich and Milton 1982; Purvis et al. 2008), allows us to estimate the Ca^2+^-binding capacity of the intracellular stores using the following equation:


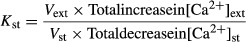
(1)

Where *V*_ext_ is the volume of the extracellular fluid, *V*_st_ is the volume of the intracellular stores, and [Ca^2+^]_ext_ is the total extracellular Ca^2+^ concentration. The extracellular volume must be corrected for the small volume of the sample occupied by platelets (Mean platelet volume is 6 fl; Fromjovich and Milton 1982), such that:


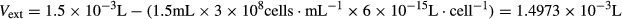
(2)

Previously published data suggest that alpha granules, dense granules, and the dense tubular system occupy 10%, 1%, and 5% of the platelet volume, respectively (Fromjovich and Milton 1982; Purvis et al. 2008). Using this information we can calculate that:


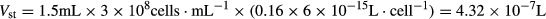
(3)

Using the measurements of the decrease in [Ca^2+^]_st_ and rise in [Ca^2+^]_ext_, K_st_ was calculated from data sets obtained from the cells of five donors coloaded with either Fura-2, Fura-4F or Fura-FF, or no cytosolic dye at all.

These data gave a mean *K*_st_ of 1043 ± 179 for Fura-2-loaded cells. There was no significant difference between the measurements of *K*_st_ made in cells loaded with Fura-4F (1040 ± 120; *P* = 0.97), Fura-FF (1066 ± 88; *P* = 0.86) or no cytosolic dye (1003 ± 102; *P* = 0.70). Given the lack of any significant difference between these results, the data were pooled to give a final estimate of *K*_st_ over the 20 data sets of 1039 ± 57 (Maximum = 1480, Minimum = 751). This estimate of *K*_st_ was then used in subsequent calculations.

### Estimation of calcium-binding capacity of the platelet cytosol

The Ca^2+^-binding capacity of the platelet cytosol was calculated by measuring the rate of increase in [Ca^2+^]_ext_ as well as the rate of decline in both [Ca^2+^]_cyt_ and [Ca^2+^]_st_ over the final 3 min of the recordings. This timeframe was chosen as the store was largely depleted at this point, such that Ca^2+^ influx into the cytosol was not the dominant Ca^2+^ flux at this time, and the rates of change in [Ca^2+^]_cyt_ and [Ca^2+^]_ext_ are both well approximated by linear functions, as shown in Figure 12. Therefore, rates of change were calculated using GraphPad Prism™ software to undertake linear regression of the data from the last 3 min of the recording.

The net Ca^2+^ flux out of the cytosol (Rate of Ca^2+^ removal into the external medium – Rate of Ca^2+^ release from the intracellular stores) was compared against the net rate of free Ca^2+^ decline in the cytosol using the following equation:


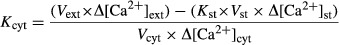
(4)

Where *K*_st_ was taken as 1039 and *V*_cyt_ was calculated as the difference between total platelet volume and *V*_st_ such that:


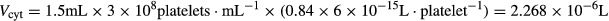
(5)

Using this value we calculated *K*_cyt_ for the cells of each of the five donors loaded with the different cytosolic dyes. These data showed that *K*_cyt_ in the presence of Fura-2 was 18,311 ± 4064. This is significantly greater than the value obtained in cells loaded with Fura-4F (8214 ± 620; *P <* 0.05), suggesting that this widely used indicator significantly adds to the Ca^2+^ buffering of platelets. Curiously, *K*_cyt_ in Fura-FF-loaded cells was not significantly different from that of cells loaded either of the other indicators (9638 ± 2219; *P* = 0.09 for Fura-2, *P* = 0.55 for Fura-4F). As the mean *K*_cyt_ obtained from Fura-FF-loaded cells is not lower than that observed with Fura-4F, the measurement of *K*_cyt_ from the medium-affinity dye is likely to be a reasonable initial estimate of the endogenous Ca^2+^-binding capacity of the platelet cytosol.

As can be observed in Figure 12, the ability of Fura-2 to act as a significant exogenous buffer in the platelet cytosol dampens the Ca^2+^ dynamics observed in all areas of the cell compared to those observed in cells loaded with Fura-4F, with the rises in [Ca^2+^]_cyt_ and [Ca^2+^]_ext_ after addition of nigericin and SERCA inhibitors being reduced to 63.3 ± 6.9% or 19.9 ± 11.2% of control, respectively, while the corresponding fall in [Ca^2+^]_st_ was reduced to 69.5 ± 6.8% of control (all *n* = 5; *P* < 0.05).

The calculated Ca^2+^-binding capacities of the cytosol and stores obtained above are both noticeably high compared to previously described values in other cells (Schwaller 2010). The measurement of *K*_st_ is also significantly higher than the Ca^2+^-binding capacity of 20 reported by Mogami et al. (1999) for the endoplasmic reticulum (ER) of pancreatic acinar cells. Our result is, however, not directly comparable due to our measurement of intracellular stores encompassing both the platelet dense tubular system (the platelet equivalent of the endoplasmic reticulum) as well as the platelet acidic organelles (Sage et al. 2011). The acidic Ca^2+^ stores are likely to encompass both the dense granule system in which Ca^2+^ is buffered by insoluble phosphate complexes, and the alpha granules, which contain high concentrations of fibrinogen, which is able to bind Ca^2+^ ions (Sato et al. 1975; Holmsen and Weiss 1979; Mihalyi 2004; Ruiz et al. 2004). Therefore it seems plausible that the Ca^2+^-binding capacity of the platelet intracellular stores is significantly higher than in the ER alone in other cell types.

The highest previously reported value for *K*_cyt_ is around 4000 in murine neuroblastoma cells (Bolsover 1986). Thus, our data suggesting an endogenous value around double this in Fura-4F-loaded platelets indicates that these cells are likely to have a very significant Ca^2+^ buffering capacity. This is perhaps not surprising given the tiny volume of platelets, which, without heavy Ca^2+^ buffering, would be exposed to the risk of developing cytotoxic increases in [Ca^2+^]_cyt_ from even the weakest stimuli. For instance, a calculation of ion fluxes into a platelet suggests that a rise in [Ca^2+^]_cyt_ from a resting level of around 100 nmol L^−1^ to 1 μmol L^−1^ would require a net accumulation of around 5.4 × 10^−21^ moles of Ca^2+^ in a 6 fl platelet. We can compare this to the current generated through a single Orai1 channel:


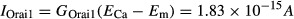
(6)

Where *G*_Orai1_ = 10 fS (Prakriya and Lewis 2003), E_m_ = −60 mV (MacIntyre and Rink 1982; Pipili 1985), and assuming [Ca^2+^]_ext_ = 1 mmol L^−1^, [Ca^2+^]_cyt_ = 100nmol L^−1^ and T = 310 K.

Using the Faraday constant we can calculate that Ca^2+^ would enter through an open Orai1 channel at 1.9 × 10^−20^ moles sec^−1^, such that cytotoxic Ca^2+^ levels could accumulate if this single channel opened for just 285 ms in the absence of any buffering, sequestration or removal. However, a quantitative analysis of the platelet proteome calculated that platelets contain around 1700 copies of the Orai1 protein (Burkhart et al. 2012). In addition, platelets also express a large number of other Ca^2+^-permeable channels with significantly larger Ca^2+^ conductances which might more easily overload the platelet with Ca^2+^. These include 4500 copies of the different isoforms of the IP_3_ receptor and around 2500 copies of other plasma membrane-based Ca^2+^ channels such as TRPC6 and P_2X1_ (Burkhart et al. 2012). Thus, without heavy Ca^2+^ buffering, the platelet would not be able to use Ca^2+^ as a second messenger without putting itself at significant risk of cytotoxic rises in [Ca^2+^]_cyt_.

The high *K*_cyt_ value also allows us to suggest a possible hypothesis for the need for pericellular Ca^2+^ recycling in platelets. The high Ca^2+^-binding capacity would be expected to significantly reduce the ability of Ca^2+^ ions to diffuse at any significant speed through the platelet cytosol, thereby slowing the triggering of Ca^2+^-dependent processes required for efficient thrombus formation, such as adhesion, granule secretion, and aggregation. Given the short time frame during which platelets encounter a damaged vessel wall, this could have significant consequences for their ability to effectively clot at a site of vascular damage. Therefore, the OCS may provide another route for rapid Ca^2+^ movement between subcompartments of the cytosol, through a Ca^2+^ tunnel, in much the same way the ER does in neurons and pancreatic acinar cells (Petersen and Verkhratsky 2007). Ca^2+^ tunneling would allow rapid triggering of the Ca^2+^-dependent events of thrombus formation while also protecting the cell from cytotoxic global increases in [Ca^2+^]_cyt_.

### Quantitative analysis of calibrated data

**Figure 13:**
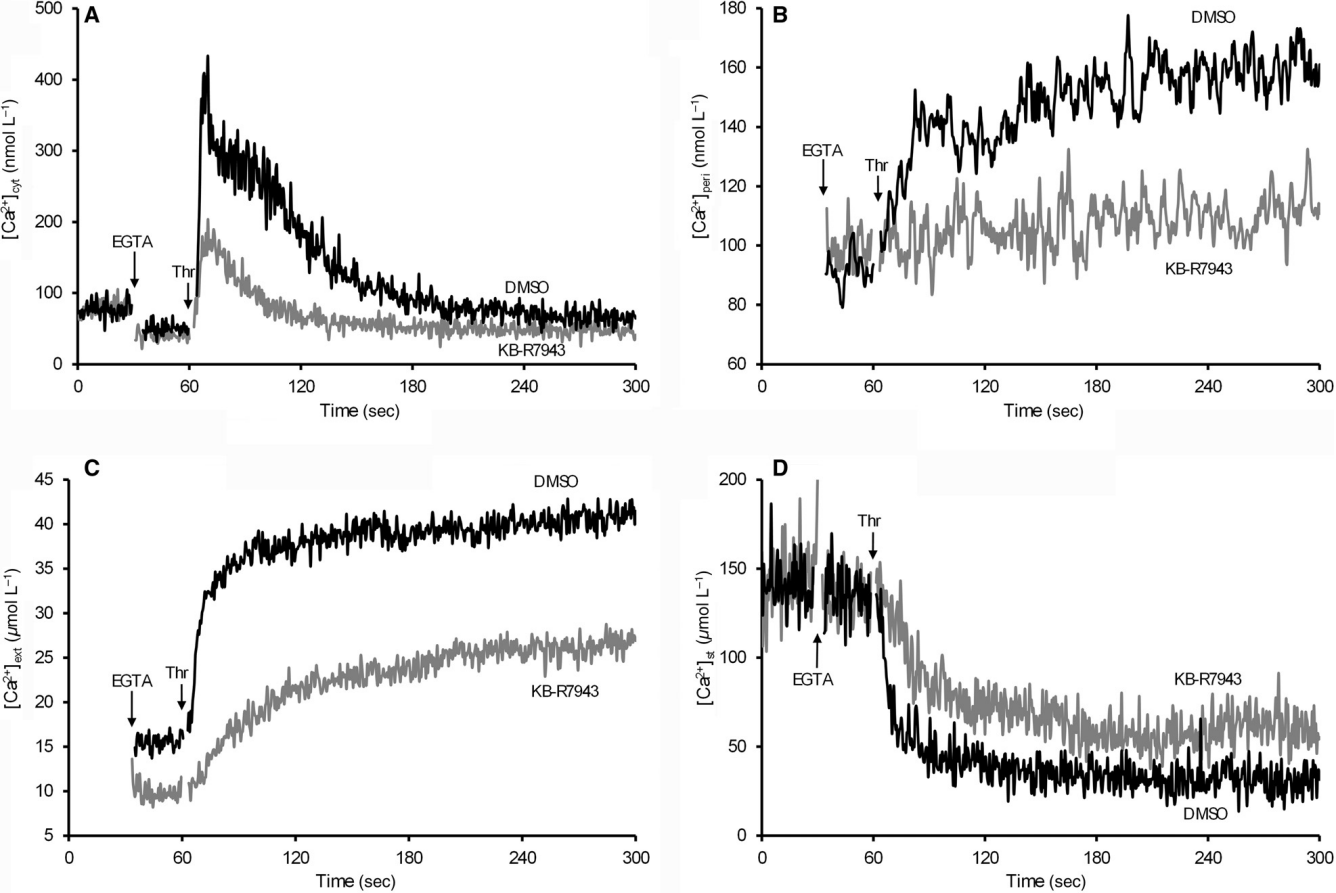
Quantitative assessment of thrombin-evoked rises in free [Ca^2+^]_cyt_, [Ca^2+^]_peri_, and [Ca^2+^]_st_ as well as the absolute increase in [Ca^2+^]_ext_ in platelets from a single donor. Fura-2- (A), FFP-18- (B) or Fluo-5N -loaded human platelets (D) or platelets suspended in supplemented Hepes-buffered saline with 2.5 μmol L^−1^ Fluo-4 (C) were pretreated with 50 μmol L^−1^ KB-R7943 or its vehicle, dimethylsulfoxide, for 2 min at 37°C. EGTA (1 mmol L^−1^) was added before the cells were stimulated with 0.5 U mL^−1^ thrombin.

To quantitatively examine the feasibility of pericellular Ca^2+^ recycling, calibrated measurements of thrombin-evoked rises in [Ca^2+^]_cyt,_ [Ca^2+^]_peri_, [Ca^2+^]_ext_, and [Ca^2+^]_st_ were made from cells from two individual donors. These results were found to be representative of individual calibrated data sets obtained throughout the study; however, as they were obtained on the same day from the same blood donation, these results could be directly compared while minimizing the risk of introducing confounding influences brought about by interindividual or interdonation variability. A set of data obtained from one of the donors is shown in Figure 13.

### Examining whether the NCX can account for the measured changes in [Ca^2+^]_ext_ and [Na^+^]_cyt_

Fluo-4 measurements showed an accumulation of extracellular Ca^2+^ in the first 10 sec poststimulation of:


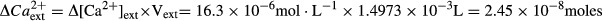
(7)

This accumulation was reduced in this donor to 56.7% of control by prior treatment with 50 μmol L^−1^ KB-R7943 (Fig. 13C); therefore, the Ca^2+^ flux through the NCX can be estimated to be 1.39 × 10^−8^ moles over this 10 sec period. If this is the Ca^2+^ removed in 10 sec from the 4.5 × 10^8^ platelets in the sample, each containing 580 copies of NCX3 (Harper et al. 2010; Burkhart et al. 2012), then we can calculate that each transporter must be transporting around 3200 Ca^2+^ ions per second. Previous measurements have suggested that Na^+^/Ca^2+^ exchangers have a maximum transport capacity of about 5000 Ca^2+^ ions per second (DiPolo and Beaugé 1996), suggesting that the exchangers are capable of this rate of removal but must be working close to full capacity. The NCX is reported to be activated by cytosolic Ca^2+^ with a *K*_0.5_ (half maximal activation) in the range 0.6–6 μmol L^−1^ (Blaustein and Lederer 1999). As the measured peak [Ca^2+^]_cyt_ in our experiments was ≈0.4 μmol L^−1^, our calculations suggest that the NCX molecules are being exposed to a microdomain of high Ca^2+^ in close proximity to the IP_3_ receptors, which allows them to rapidly remove Ca^2+^ at near saturating rates. This would be consistent with our suggested model of the NCX's being selectively localized close to the principal Ca^2+^ release site in the DTS in the platelet membrane complex.

Previous work by Stamouli et al. (1993) reported that thrombin evoked a rise in [Na^+^]_cyt_ from a resting level of 5 mmol L^−1^ to 27 mmol L^−1^ after stimulation. Calculations were therefore performed to examine whether the measured rate of net Ca^2+^ removal through the NCX into the extracellular medium could account for the Na^+^ accumulation observed in thrombin-stimulated platelets. Given the 3:1 stoichiometry of the NCX, this would equate to 4.16 × 10^−8^ moles of Na^+^ entering the platelet cytosol which would elicit a rise in [Na^+^]_cyt_ of:


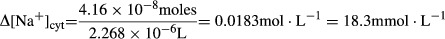
(8)

Thus, the NCX could account for the majority of the [Na^+^]_cyt_ rise observed in thrombin-stimulated platelets. Indeed, this is consistent with our own data that shows that blocking NCXs with KB-R7943 inhibits thrombin-evoked rises in [Na^+^]_cyt_ to around 15% of control values (Fig. 1D). Therefore, our data suggest that the NCX is capable of eliciting the Na^+^ flux previously observed.

### The pericellular Ca^2+^ increase is not due to nonspecific increases in the free Ca^2+^ concentration in the bulk extracellular medium

Our measurements of thrombin-evoked increase in extracellular Ca^2+^ measure the total Ca^2+^ removed from the platelet (see Methods). Although we have heavily buffered the extracellular medium with 1 mmol L^−1^ EGTA, the addition of further Ca^2+^ upon agonist stimulation will cause a small increase in the free Ca^2+^ concentration of the extracellular medium. We therefore used the MaxChelator program (http://maxchelator.stanford.edu) to calculate the increase in free [Ca^2+^]_ext_ that would be expected in response to these agonist-evoked changes in total Ca^2+^.

The HBS used in our experiments contained 200 μmol L^−1^ CaCl_2_ to prevent platelet store depletion on resuspension, to this we added 1 mmol L^−1^ EGTA in all experiments which would chelate the vast majority of the free Ca^2+^ ions. When calibrating the Fluo-4 salt measurements of total [Ca^2+^]_ext_, the fluorescence taken from a platelet-free sample of supplemented HBS was taken as the baseline fluorescence. We then created a calibration curve through the addition of Ca^2+^ to the medium to give known total [Ca^2+^]_ext_. This calibration curve was then used in a linear regression fit to calibrate the additional Ca^2+^ added to the medium following agonist stimulation. As shown in Figure 13C, there was a basal value of 15.7 μmol L^−1^ (which gives a total [Ca^2+^]_ext_ of 215.7 μmol L^−1^ after taking account the 200 μmol L^−1^ Ca^2+^ that was preadded to the medium). After thrombin stimulation for 10 sec, the measured [Ca^2+^]_ext_ increased to 32.1 μmol L^−1^ (thus a final concentration of 232.1 μmol L^−1^). Utilizing the two chelators, two metal version of MaxChelator with the standard conditions of T = 37°C, pH = 7.4, Ionic concentration = 0.154N, EGTA = 0.001 mol L^−1^, Mg = 0.001 mol L^−1^, and ATP = 0 mol L^−1^, the calculated increase in total extracellular Ca^2+^ concentration would lead to an increase in free [Ca^2+^]_ext_ of 2.9 nmol L^−1^. This is much smaller than the 31.1 nmol L^−1^ increase in the pericellular free Ca^2+^ concentration observed over the same time period, suggesting that our measurement is due to a genuine accumulation of Ca^2+^ specifically in the pericellular region, rather than being an artifact of a generalized increase in free Ca^2+^ concentration through the bulk extracellular medium.

### Can the measured pericellular calcium concentrations elicit recycling?

**Figure 14:**
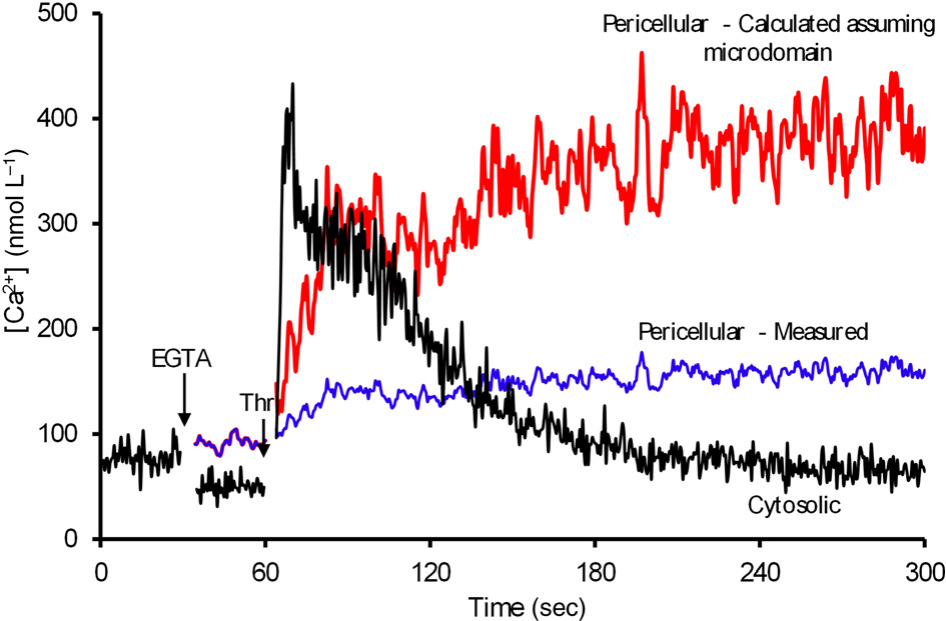
Comparison of thrombin-evoked rises in free [Ca^2+^]_cyt_ and [Ca^2+^]_peri_ in platelets from a single donor. Fura-2- or FFP-18-loaded human platelets were suspended in supplemented Hepes-buffered saline. EGTA (1 mmol L^−1^) was added before the cells were stimulated with 0.5 U mL^−1^ thrombin. The measured changes in [Ca^2+^]_cyt_ are shown in black, the measured changes in [Ca^2+^]_peri_ are shown in blue, and the estimated changes in [Ca^2+^]_peri_ assuming microdomains of the size determined from imaging experiments are shown in red.

To be able to elicit pericellular Ca^2+^ recycling back into the platelet through Ca^2+^-permeable ion channels there must be an electrochemical gradient created between the pericellular region and the cytosol. Given the platelet membrane potential does not significantly depolarise during thrombin stimulation (MacIntyre and Rink 1982; Pipili 1985), this principally involves maintaining a concentration gradient between these two compartments. To examine whether there is a noticeable gradient formed, the results from the pericellular and cytosolic Ca^2+^ measurements from Figure 13 were overlaid on the same scale (Fig. 14). This analysis suggested that there was not a significant Ca^2+^ concentration gradient formed for pericellular recycling during much of the platelet Ca^2+^ signal.

However, as described above, we observed the formation of microdomains of pericellular Ca^2+^ in specific subregions of the cell (Fig. 10) suggesting that rather than being equally distributed around the platelet, only a small pericellular subregion may experience a rise in free [Ca^2+^]_peri_. As the pericellular Ca^2+^ signal is not equally distributed, but is principally observed in microdomains of the cell, the calibrated FFP-18 data will underestimate the amplitude of the free [Ca^2+^]_peri_ as much of the FFP-18 will see no significant increase in [Ca^2+^]. Given that the size of the pericellular Ca^2+^ microdomains observed (Fig. 10) closely correlated with the hotspots in FFP-18 fluorescence (Fig. 11C; presumably the platelet membrane complex), we scaled the agonist-evoked increases in free [Ca^2+^]_peri_ to reflect the pericellular Ca^2+^ signal that might be generated in this subregion. As discussed above, 23.2% of the total FFP-18 fluorescence was found in this subregion (Fig. 11). Using this assumption that the pericellular Ca^2+^ signal is compartmentalized and only experienced by the FFP-18 localized in this region, it was possible to show that there would be a Ca^2+^ concentration gradient that could be used to directly drive a Ca^2+^ influx that could maintain the cytosolic Ca^2+^ signal from 10 sec after thrombin stimulation until the end of the recording, creating the small plateau observed in the cytosolic Ca^2+^ measurement between 10 and 40 sec after thrombin addition (Fig. 14). Thus, our data support the plausibility of pericellular recycling, but our quantitative analysis suggests that the microdomain of pericellular Ca^2+^ accumulation observed in our single cell recording is essential to this process.

### A quantitative analysis of how blocking pericellular recycling by pretreatment with KB-R7943 affects platelet calcium handling

#### How can calcium recycling affect the initial peak in the cytosolic calcium signal?

The above analysis fails to explain how KB-R7943 pretreatment causes a reduction in the initial spike in [Ca^2+^]_cyt_ seen for the first 10 sec after thrombin stimulation in both Figures 1B, 13A. The Fluo-5N data indicate a difference in the rate of depletion of Ca^2+^ from the intracellular stores during this time period (Figs. 3, 4D). Interestingly, this period also correlates well with the transient spike in IP_3_ concentration previously observed in thrombin-stimulated platelets (Rittenhouse and Sasson 1985), suggesting that this initial phase of the Ca^2+^ signal is driven by the opening of IP_3_ receptors. We therefore performed a linear regression analysis using GraphPad Prism™ software to determine the rate of depletion of the Ca^2+^ stores during the first 10 sec of the thrombin-evoked response to examine whether this could account for the difference in the [Ca^2+^]_cyt_ responses seen between the control and KB-R7943-treated cells. This analysis revealed that the rates of depletion of the intracellular stores in DMSO-treated (5.957 μmol L^−1^ sec^−1^) and KB-R7943-treated cells (3.365 μmol L^−1^ sec^−1^) were significantly different (*P* < 0.0001). Using the calculated volume of the intracellular stores (4.32 × 10^−7^ L) as well as *K*_st_ (1039), it was possible to use these data to estimate the total amount of Ca^2+^ released per second from the stores using the following equation:


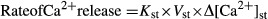
(9)

The DMSO-treated cells released 2.67 nanomoles sec^−1^ in comparison with the 1.51 nanomoles sec^−1^ released by the KB-R7943-pretreated cells. If we assume that all the released Ca^2+^ accumulates in the cytosolic volume (defined above as 2.27 × 10^−6^ L) and is subject to the same Ca^2+^ buffering conditions previously calculated (*K*_cyt_ = 18,311 in Fura-2-loaded cells), we can then calculate the peak of the Ca^2+^ spike expected after 10 sec of store depletion under both conditions using the following equation:


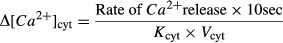
(10)

These equations predict a rise in [Ca^2+^]_cyt_ of 693 nmol L^−1^ for the control cells and a rise of only 392 nmol L^−1^ for the KB-R7943-pretreated cells. These values are in reasonable agreement with the rises in [Ca^2+^]_cyt_ measured in Fura-2-loaded cells (Fig. 13A) of 350 nmol L^−1^ and 150 nmol L^−1^ for the control and NCX-inhibited cells, respectively. The differences between the calculated and measured values of [Ca^2+^]_cyt_ are likely to be due to the calculated values failing to take account of the effect of Ca^2+^ removal from the cell and sequestration back into the intracellular stores.

As interfering with pericellular Ca^2+^ recycling in ways other than NCX inhibition, such as blocking plasma membrane Ca^2+^ channels (Fig. 7) or buffering the pericellular signal with extracellular BAPTA (Fig. 8) affects the release of Ca^2+^ from intracellular stores, these data suggest that Ca^2+^ reentry from the pericellular region has a role in facilitating Ca^2+^ release from intracellular stores. While there may be no observable Ca^2+^ gradient at this time between the measured rises in [Ca^2+^]_cyt_ and [Ca^2+^]_peri_, there may be a local effect whereby Ca^2+^ recycles into subregions of the platelet cytosol in which the nearby IP_3_ receptors are inactive. Ca^2+^ recycling into these regions may trigger the opening of these Ca^2+^ release channels through a Ca^2+^-induced Ca^2+^ release (CICR) mechanism, allowing maximal Ca^2+^ release from the intracellular stores. Consistent with this possibility, research in murine cardiac myocytes has demonstrated that the NCX can play a role in ensuring synchronous Ca^2+^ release from dyadic release sites (Neco et al. 2010).

**Figure 15:**
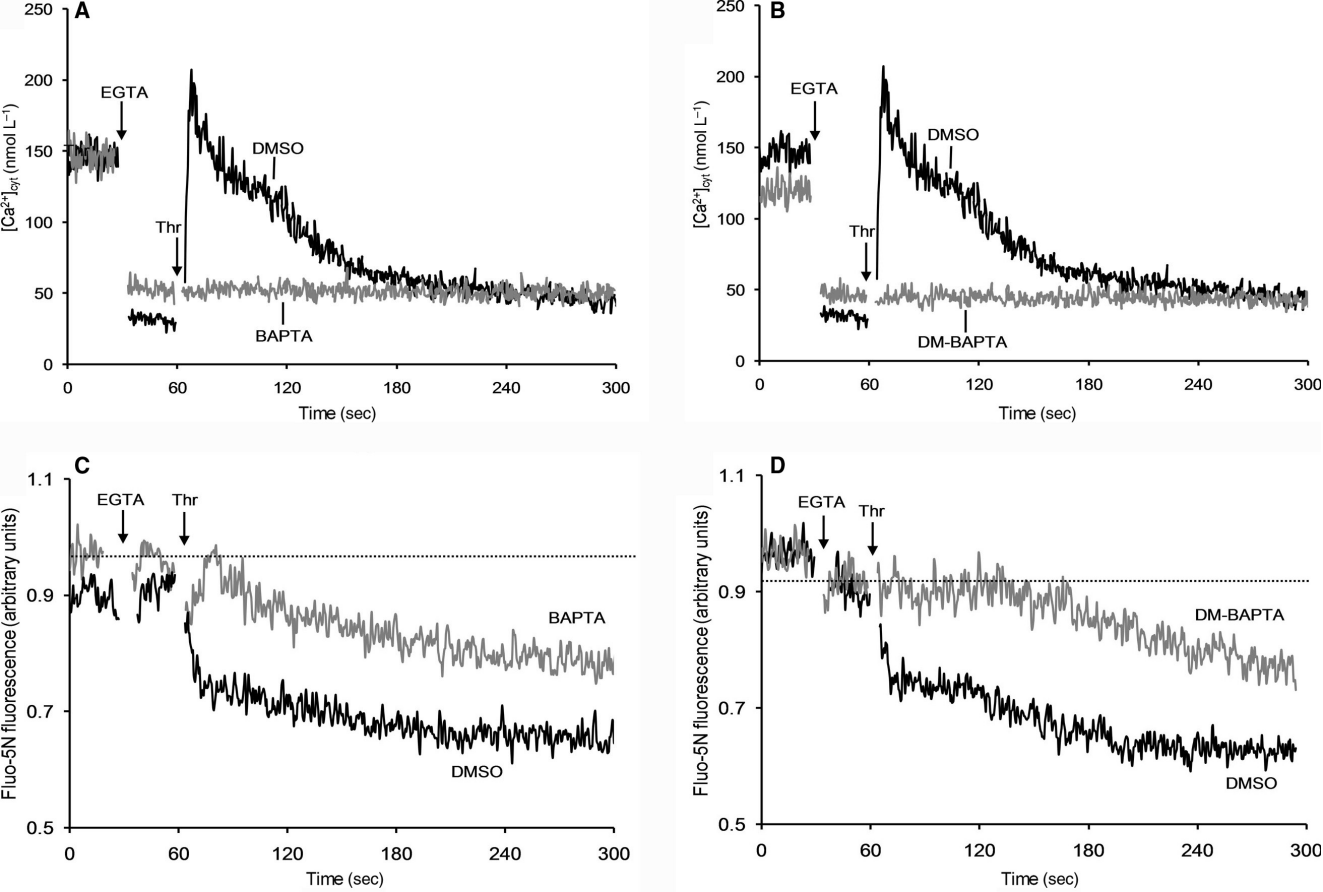
Effects on release of Ca^2+^ from intracellular stores of buffering rises in [Ca^2+^]_cyt_ by loading platelets with the Ca^2+^ chelators BAPTA or dimethyl-BAPTA (DM-BAPTA). Fura-2- (A, B) or Fluo-5N-loaded (C, D) human platelets were suspended in supplemented Hepes-buffered saline and preincubated with either 30 μmol L^−1^ BAPTA/AM (A, C), 30 μmol L^−1^ DM-BAPTA/AM (B, D) or an equal volume of their vehicle (dimethylsulfoxide) for 10 min at 37°C. 1 mmol L^−1^ EGTA was added before the cells were stimulated 30 sec later with 0.5 U mL^−1^ thrombin. Fluo-5N results were corrected for the small autofluorescence of DM-BAPTA/AM. A and B have been reproduced from Figure 3 (E and F) to allow direct comparison of effects of the chelators on [Ca^2+^]_cyt_ and [Ca^2+^]_st_.

We (Sage et al. 2011) and others (Somasunduram et al. 1997; van Gorp et al. 2002) have previously presented initial evidence for a role of CICR in platelets; however, we have further investigated whether CICR could play a role in potentiating thrombin-evoked Ca^2+^ release from intracellular stores. This was examined by preincubating Fluo-5N-loaded platelets with BAPTA/AM or DM-BAPTA/AM. Loading with either Ca^2+^ chelator prevented any significant rise in [Ca^2+^]_cyt_ in response to thrombin stimulation (Fig. 15A and B; 9.2 ± 3.7% or 1.5 ± 1.2% of control for BAPTA or DM-BAPTA loading, respectively; *n* = 5 or 6, respectively; both *P* < 0.05). Both of these compounds also markedly reduced the thrombin-evoked decrease in [Ca^2+^]_st_ (Fig. 15C and D; 52.5 ± 6.4% and 17.1 ± 4.0% of control for BAPTA or DM-BAPTA, respectively; *n* = 5 or 6, respectively; both *P* < 0.05). Both chelators caused a marked reduction in the net Ca^2+^ release over the first 15 sec of the response to thrombin. The magnitude of inhibition of net thrombin-evoked Ca^2+^ release by BAPTA or DM-BAPTA loading appeared to be inversely correlated with the K_d_ of the buffer concerned, with DM-BAPTA having a lower effective K_d_ than BAPTA (Pethig et al. 1989). These results suggest that in the absence of changes in [Ca^2+^]_cyt_, a rise in IP_3_ concentration elicits only weak Ca^2+^ release from the intracellular stores. Therefore, pericellular recycling may play a role in ensuring the rapid recruitment of all IP_3_ receptors to trigger maximal Ca^2+^ release from the intracellular stores.

Although Neco et al. (2010) demonstrated that the NCX may play a role in assisting synchronous Ca^2+^ release in cardiac myocytes, in their model this was thought to be associated with Ca^2+^ entry through reverse-mode exchange. In contrast, our model suggests that the NCX must work in forward mode for the following reasons: (i) As pointed out above, the cytosolic Na^+^ accumulation and Ca^2+^ efflux observed are only possible if the NCX is working in forward mode at near full capacity, (ii) Unlike cardiac myocytes, platelets are nonexcitable cells and so there is only a minimal membrane depolarization observable upon agonist stimulation (MacIntyre and Rink 1982; Pipili 1985) and thus it is unlikely the NCX will reach its reversal potential, and (iii) The measured Ca^2+^ concentration in the pericellular region is around 0.1% of the extracellular Ca^2+^ concentration required for half maximal activity of the mammalian NCX in reverse mode (100–300 μmol L^−1^; Blaustein and Lederer 1999). Therefore, platelets may use the NCX in a different manner to achieve the same function as in cardiac myocytes, with the NCX functioning to provide a Ca^2+^ source from which Ca^2+^ may then enter into inactive subregions through Ca^2+^-permeable channels to trigger IP_3_ receptor activation. A synchronous triggering of Ca^2+^ release would make functional sense in ensuring rapid and large-scale depletion of the platelet intracellular Ca^2+^ stores to trigger the rises in [Ca^2+^]_cyt_ required to ensure rapid adherence and aggregation on damaged blood vessel walls in the presence of blood flow.

#### How does pretreatment with KB-R7943 affect calcium fluxes between platelet compartments?

Using the measured changes in [Ca^2+^]_st_, [Ca^2+^]_cyt_, and [Ca^2+^]_ext_ compared to basal measurements prior to stimulation (Fig. 13), along with previous measurements of the platelet compartment volumes (Fromjovich and Milton 1982) and of the calcium-binding capacities (*K*_st_ and *K*_cyt_; Fig. 12), it is possible to calculate the changes in the amount of Ca^2+^ in each of these compartments after thrombin addition using the following equations:


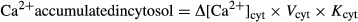
(11)


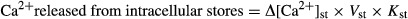
(12)


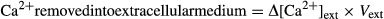
(13)

**Figure 16:**
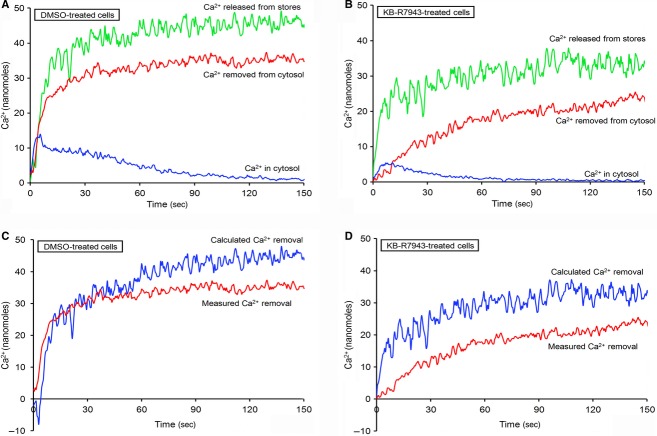
Quantitative analysis of platelet Ca^2+^ handling in control and KB-R7943-treated platelets. (A, B) Using the data from Fig. 13, the amount of Ca^2+^ released from intracellular stores, accumulated in the cytosol, and removed from the cell across the plasma membrane was calculated and plotted as a function of time for cells pretreated with dimethylsulfoxide (DMSO; Control; A) or 50 μmol L^−1^ KB-R7943 (B) following stimulation with 0.5 U mL^−1^ thrombin in a Ca^2+^-free medium. (C, D). The Ca^2+^ that was measured accumulating in the extracellular medium using Fluo-4 was compared to the amount of Ca^2+^ that would have been expected to be removed from the cytosol using the measurements of intracellular Ca^2+^ store depletion and cytosolic Ca^2+^ rise. These calculations were performed for both cells pretreated with DMSO (Control; C) and those pretreated with 50 *µ*mol L^−1^ KB-R7943 (D) following stimulation with 0.5 U mL^−1^ thrombin in a Ca^2+^-free medium.

Using these calculated changes it is possible to examine whether there are differences in the manner Ca^2+^ is handled by control cells and those in which pericellular recycling is inhibited by pretreatment with KB-R7943 (Fig. 16). This analysis demonstrated that there are differences in the time courses of Ca^2+^ changes in each of the platelet subcompartments.

In KB-R7943-treated cells, it can be seen that following stimulation by thrombin there was an immediate release of Ca^2+^ from the intracellular stores, followed a short time later by a rise in the amount of Ca^2+^ in the cytosol and then, a few seconds later, this was followed by Ca^2+^ accumulation in the extracellular medium (Fig. 16B). This pattern of events suggests that in these cells Ca^2+^ leaves the intracellular stores and accumulates in the cytosolic compartment before being removed across the plasma membrane into the extracellular medium. However, in the control cells this pattern was not observed, with all three compartments showing closely correlated changes in Ca^2+^ content in the first 10 sec after stimulation (Fig. 16A). The kinetics of these Ca^2+^ fluxes suggests a tight coupling of Ca^2+^ release from the intracellular stores and Ca^2+^ removal by the NCX, such that there is no distinguishable difference in latency between the onset of Ca^2+^ release from the stores, entry into the cytosol and removal into the extracellular medium in control cells. This close coupling of Ca^2+^ fluxes across membranes would be consistent with our proposal of a linked Ca^2+^ exchange at the platelet membrane complex, where the membranes of the dense tubular system (DTS) and OCS are intimately associated (White 1972). This linked transport would also be consistent with the microdomains of high [Ca^2+^] observed by single cell imaging of extracellular Ca^2+^ (Fig. 10). Pretreatment with KB-R7943 therefore may abolish the thrombin-evoked rise in [Ca^2+^]_peri_ (Fig. 13B) by interfering with this tight linkage of Ca^2+^ release and removal at this microdomain so that Ca^2+^ has to translocate further through the cytosol to other locations to be removed across the plasma membrane, such that there are distinct latencies between the onset of Ca^2+^ release, accumulation in the cytosol and removal to the extracellular medium.

#### Why there is no observable rise in [Ca^2+^]_peri_ in KB-R7943 pretreated cells?

The data presented in Fig. 13 (as well as in Fig. 1C) show that KB-R7943 treatment only partially inhibits Ca^2+^ removal into the extracellular medium (Fig. 13C), but completely abolishes the thrombin-evoked rise in [Ca^2+^]_peri_. As some Ca^2+^ is being removed in the presence of the NCX inhibitor, Ca^2+^ must be entering the pericellular region, suggesting that the Ca^2+^ removed from the cell must be bound by some buffer associated with the platelet surface in addition to the exogenous EGTA (which is accounted for in the Fluo-4 calibration). This is consistent with previous work by Brass and Shattil (1982), which demonstrated that the platelet extracellular surface contains at least two high-affinity Ca^2+^ binding sites. Furthermore, the experiments with chondroitinase demonstrated that digestion of the platelet glycocalyx increased the amount of Ca^2+^ measured in the extracellular medium by Fluo-4 but reduced the amount of Ca^2+^ detected by FFP-18 in the pericellular region, which suggests a possible role for the glycocalyx in binding Ca^2+^ and holding it within the pericellular region (Fig. 9). Thus, Ca^2+^ buffering by the platelet surface may also work to buffer a proportion of the Ca^2+^ removed from the platelets preventing rises in [Ca^2+^]_peri_ at slower rates of Ca^2+^ removal, such that no change is observed by the FFP-18.

If Ca^2+^ is being buffered in the pericellular region, then the amount of Ca^2+^ measured entering the extracellular medium by Fluo-4 should be less than that would be predicted from our measurements of the changes in the amounts of Ca^2+^ found in the cytosol and intracellular stores, with the expected amount of Ca^2+^ being removed being calculated from the following equation:


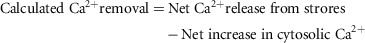
(14)

The Ca^2+^ removal measured using Fluo-4 and calculated from the Fura-2 and Fluo-5N traces was compared for both DMSO- (Fig. 16C) and KB-R7943-treated (Fig. 16D) platelets. The control cells show a close match between the measured and calculated Ca^2+^ removals for the first 45 sec after stimulation by thrombin (Fig. 16C). This shows that the model is capable of accurately accounting for all the Ca^2+^ flux in the system and provides an independent validation of the previously calculated parameters, *K*_st_ and *K*_cyt_.

However, beyond the first 45 sec after stimulation there is a gradual divergence of the calculated and measured Ca^2+^ removals suggesting that Ca^2+^ begins to be buffered in the extracellular medium by an additional buffer. This appears to correlate well with the point at which Ca^2+^ removal starts to slow due to the [Ca^2+^]_cyt_ beginning to fall back toward basal levels (Fig. 13A). Thus, when Ca^2+^ removal is occurring at a high rates, the rapid accumulation of Ca^2+^ in the pericellular region allows effective recycling such that Ca^2+^ is not effectively buffered, whereas at slower rates of translocation, Ca^2+^ recycling is less effective giving time for Ca^2+^ to be bound to the pericellular buffers. In contrast, KB-R7943-treated cells show a large discrepancy from the time of stimulation by thrombin, suggesting that the loss of high capacity Ca^2+^ transport by the NCX allows the remaining Ca^2+^ that is removed to be adequately buffered in the pericellular region.

Interestingly, both KB-R7943- and DMSO-treated cells show a similar difference between the measured and calculated Ca^2+^ removals (8.8 and 10.2 nanomoles, respectively) at the end of the recording period, suggesting that this might represent the total capacity of the additional extracellular Ca^2+^ buffer. The final calculated values are 137.5% and 129.5% of the measured values in KB-R7943- and DMSO-treated cells, respectively, which is similar to the 128.7% increase in the Fluo-4 signal observed in chondroitinase-treated cells. Further experiments will be needed to confirm these initial analyses, but the results are consistent with a role for the glycocalyx in buffering Ca^2+^ in the pericellular region.

#### Could removal of Ca^2+^ into the open canalicular system account for the increase in Ca^2+^ concentration observed in the pericellular region?

**Figure 17:**
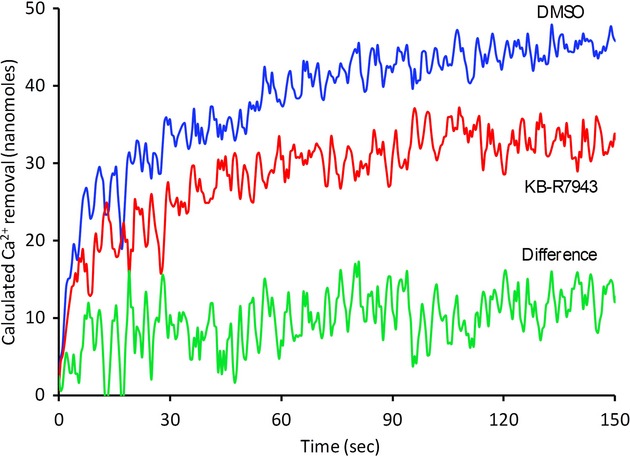
Estimation of Ca^2+^ removal involved in eliciting the pericellular Ca^2+^ signal. The calculated Ca^2+^ removal data for dimethylsulfoxide- and KB-R7943-treated cells from Fig. 16 (red and blue lines) is overlaid here and the calculated difference between these is plotted.

The above results suggest that the calculated Ca^2+^ removal data may more accurately resemble the amount of Ca^2+^ translocating across the platelet plasma membrane than the removal measured using extracellular Fluo-4. As KB-R7943-treated cells show no observable pericellular signal, the difference in the calculated Ca^2+^ removed from DMSO- and KB-R7943-treated cells should represent a good estimate of the amount of Ca^2+^ responsible for generating the pericellular signal. As shown in Figure 17, this calculation indicates a gradual accumulation of an additional 10.8 nanomoles over the recording period (150 sec), giving an average rate of removal of 72.2 picomoles sec^−1^ during this time. As the OCS is continuous with the bulk extracellular medium, we assumed that the pericellular Ca^2+^ concentration would reflect the rate of Ca^2+^ removal as the bulk extracellular medium can rapidly exchange with the OCS. This assumption is supported by our experiments showing that EGTA addition to FFP-18-loaded cells suspended in high external Ca^2+^ results in a rapid fall in the FFP-18 fluorescence ratio to a new steady value (data not shown) suggesting that there is good exchange between these two compartments.

If 72.2 picomoles of Ca^2+^ was introduced into the volume of the total OCS found in our samples (6% of the platelet volume; Fromjovich and Milton 1982; 0; 06 × 4.5 × 10^8^ platelets × 6 fl = 1.62 × 10^−7^ L) this would be expected to cause the total [Ca^2+^] of the OCS ([Ca^2+^]_OCS_) to increase by 446 μmol L^−1^. If we assume that the OCS contents are identical to the rest of the extracellular medium, we can again use the Maxchelator program to calculate the expected increase in free [Ca^2+^]_OCS_ that increasing the total Ca^2+^ concentration from 200 μmol L^−1^ to 646 μmol L^−1^ would elicit (using the following constants: T = 37°C, pH = 7.4, Ionic concentration = 0.154N, EGTA = 0.001 mol L^−1^, Mg = 0.001 mol L^−1^, and ATP = 0 mol L^−1^). This indicates that the calculated rate of Ca^2+^ entry into the OCS would be expected to increase the free [Ca^2+^]_OCS_ from 29 nmol L^−1^ to 197 nmol L^−1^. This fits reasonably well with our FFP-18 measurements of [Ca^2+^]_peri_ which increase from a basal level of 91 nmol L^−1^ to 141 nmol L^−1^ after 30 sec of thrombin stimulation (Fig. 13B). Thus, it is possible to reconcile much of the measured Ca^2+^ signaling data with our proposed model of Ca^2+^ recycling.

The above mathematical analysis indicates that our measured data can be reconciled with the hypothesis that platelet pericellular Ca^2+^ recycling potentiates thrombin-evoked rises in [Ca^2+^]_cyt_ in two ways: first, by potentiating Ca^2+^ release from the intracellular stores through a process of CICR and then later on, by providing a secondary source of Ca^2+^ to enter the cytosol. This analysis provides a better understanding of the likely mechanisms of thrombin-evoked platelet Ca^2+^ signaling and should provide a basis for guiding the design of future experiments to attempt to better understand how this process occurs.

The Fluo-4 imaging experiments showed that rises in pericellular Ca^2+^ concentration begin in a specific microdomain of the cell (Fig. 10) and we believe that this might be at the platelet membrane complex (White 1972). The numerical analysis presented here supports this conclusion, as the differences between the kinetics of Ca^2+^ release and removal in control and KB-R7943-treated platelets suggest that the NCX is likely to be closely associated with the IP_3_ receptor. The likely involvement of the membrane complex is further supported by the calculations showing that the NCX must be working at near full capacity to account for the observed Ca^2+^ removal from the cells, even though the measured [Ca^2+^]_cyt_ is below the level required for half maximal activation, suggesting that the NCX must be exposed to a microdomain of high [Ca^2+^]_cyt_ such as would be expected to occur with a close association between the IP_3_ receptor and the NCX at the membrane complex.

Finally, by reexamining our data in a quantitative manner we have been able to provide initial evidence for a possible role for the glycocalyx in controlling the duration of Ca^2+^ signaling through its ability to buffer pericellular Ca^2+^ rises. The analysis also suggests that the glycocalyx could also provide pericellular Ca^2+^ recycling with a threshold-like property, whereby Ca^2+^ removed slowly following low-level stimulation may be buffered by the glycocalyx and therefore elicit no further effect, while high-level stimulation leads to rapid Ca^2+^ removal which is able to overcome buffering by the glycocalyx and so recycle, leading to more prolonged platelet activation.

## Discussion

In this study, we have presented evidence that upon activation by thrombin, platelets accumulate Ca^2+^ in a pericellular region. Stimulation of platelets with the physiological agonist thrombin evoked a rise in [Ca^2+^]_ext_ and [Ca^2+^]_peri_ as detected with extracellular Fluo-3 or Fluo-4, or the near-membrane indicator FFP-18. Single cell imaging of thrombin-evoked rises in [Ca^2+^]_ext_ indicated that these occurred at discrete sites within the boundaries of the cells, consistent with Ca^2+^ accumulation within the open canalicular system, a system of tubular invaginations of the platelet plasma membrane analogous to the transverse tubules of striated muscle (White 1972). Imaging of FFP-18 fluorescence was also consistent with the location of this indicator in the OCS. Thrombin-evoked rises in [Ca^2+^]_ext_ and [Ca^2+^]_peri_ were reduced or abolished by the NCX inhibitors SN-6 or KB-R7943 and by removal of extracellular Na^+^, indicating that these changes were largely generated by Ca^2+^ export on the NCX. NCX inhibition paradoxically inhibited thrombin-evoked rises in [Ca^2+^]_cyt_ in the absence as well as in the presence of extracellular Ca^2+^ and also inhibited the secretion of autocoids from dense granules. Similar effects on [Ca^2+^]_cyt_ have been reported in studies using other NCX inhibitors (Hunyady et al. 1987; Jy and Haynes 1987) and inhibitors of the plasma membrane Ca^2+^-ATPase (Jones et al. 2010). We therefore hypothesized that Ca^2+^ exported across the plasma membrane by the NCX may recycle back into the cytosol via Ca^2+^-permeable ion channels.

Consistent with this hypothesis, we have shown that interfering with the generation of the pericellular Ca^2+^ signal leads to a reduction in both the thrombin-evoked rise in [Ca^2+^]_cyt_ as well as dense granule secretion, whether this be by inhibition of the NCX, reducing the accumulation of Ca^2+^ in the pericellular region by treatment with chondroitinase, BAPTA or dimethyl-BAPTA, or by reducing the reentry of Ca^2+^ back into the cytosol by inhibition of Ca^2+^-permeable ion channels. Although it is possible that the effects of any individual treatment could be nonspecific, the agreement between the diverse data sets obtained from a range of different experimental manipulations of the platelet Ca^2+^ signaling system strongly suggests the presence of a pericellular Ca^2+^ recycling system. While the NCX inhibitors used in this study have been reported to have nonspecific effects in other cell types, our previous studies have shown that the effects of these compounds on platelet Ca^2+^ signaling are not mediated through nonspecific actions on several components of the platelet Ca^2+^ signaling toolkit, including the SOC, receptors for the autocoids released from dense granules, and the IP_3_ receptor (Harper and Sage 2007; Harper et al. 2009b).

Ca^2+^ recycling from the pericellular region appeared to potentiate the thrombin-evoked rise in [Ca^2+^]_cyt_ by at least two mechanisms: Facilitating Ca^2+^ store depletion and a direct effect of the recycled Ca^2+^ to maintain the elevated [Ca^2+^]_cyt_. The initial effect appeared to be related to pericellular Ca^2+^ recycling increasing the rate of Ca^2+^ release from the intracellular stores, as significantly inhibiting pericellular recycling in a number of ways (inhibiting the NCX using KB-R7943 or by removal of extracellular Na^+^, buffering pericellular Ca^2+^ rises with BAPTA or strong inhibition of the thrombin-evoked increase in plasma membrane Ca^2+^ permeability) all caused a similar inhibition in thrombin-evoked Ca^2+^ store depletion. A later effect appeared to be the direct maintenance of the [Ca^2+^]_cyt_ at raised levels by the recycled Ca^2+^, creating the plateau phase observed after the initial spike. This interpretation is supported by quantitative analysis of calibrated data sets obtained from a single donor.

Although the data presented here provide significant support for a Ca^2+^ recycling system in human platelets, there remain a number of questions about the cellular mechanisms underlying this system as well as its physiological relevance. One challenge to the model described above is the possibility that the Ca^2+^ accumulated in the pericellular space is not removed from the cytosol by the NCX but is the result of the exocytosis of Ca^2+^ stored in dense granules. Although at present we cannot definitively exclude this possibility, we favor the hypothesis that the NCX is the source of the Ca^2+^ accumulated pericellularly for the following reasons. First, the NCX inhibitor KB-R7943 abolished the creation of the pericellular Ca^2+^ signal, but only slowed and did not abolish dense granule secretion suggesting that dense granule secretion and the pericellular signal are not causally related. Second, previous work on platelets from Hermansky–Pudlak syndrome patients, whose platelets lack dense granules, has shown that the deficit in the thrombin-evoked Ca^2+^ signal can be reversed by the addition of exogenous adenine nucleotides (Lages and Weiss 1999). These data suggest that the Ca^2+^ stored within the dense granules is not required for agonist-evoked Ca^2+^ signaling and it therefore seems unlikely that Ca^2+^ within the granule is required for recycling back into the platelet cytosol.

A second question concerns why Ca^2+^ that is prevented from being removed from the platelet cytosol via the NCX (and the PMCA) is less able to influence thrombin-evoked dense granule secretion and rises in [Ca^2+^]_cyt_ than Ca^2+^ removed from the cytosol and then recycled back into the cell. In other cells in which a Ca^2+^ recycling system has been demonstrated, such as arterial smooth muscle cells (Nazer and van Breemen 1998) and cardiac myocytes (Orchard et al. 2009), the answer lies with the creation of a junctional membrane complex in which Ca^2+^ diffusion is physically restricted, preventing effective communication between a cytosolic microdomain and the bulk cytosol.

**Figure 18:**
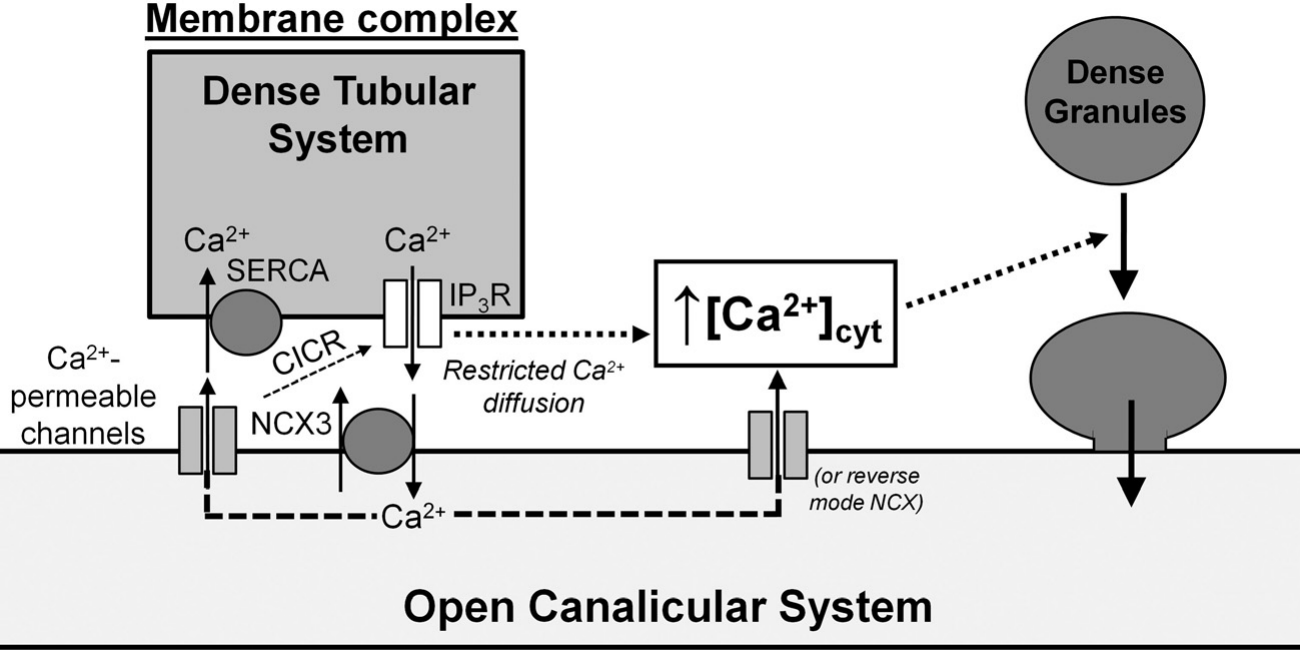
Model outlining a pericellular Ca^2+^ recycling system in thrombin-stimulated human platelets. Protease activated receptor activation elicits a rapid rise in IP_3_ (not shown), which triggers Ca^2+^ release from the dense tubular system by activation of IP_3_ receptors. This triggers the initial thrombin-evoked rise in [Ca^2+^]_cyt_. Previous work has demonstrated that this initial Ca^2+^ rise is affected by Ca^2+^ reuptake by SERCA (Sage et al. 2011). NCX-mediated Ca^2+^ removal allows the accumulation Ca^2+^ within the open canalicular system. The elevation of pericellular Ca^2+^ can create a concentration gradient which causes this Ca^2+^ to recycle back into the cytosol through Ca^2+^-permeable ion channels (or possibly reverse mode NCX). This recycled Ca^2+^ reinforces the initial Ca^2+^ rise through two mechanisms: (1) recycled Ca^2+^ triggers further calcium release via Ca^2+^-induced Ca^2+^ release therefore eliciting a rapid rise in the [Ca^2+^]_cyt_; (2) recycled Ca^2+^ directly contributes to the maintenance of the thrombin-evoked Ca^2+^ signal by moving through Ca^2+^- permeable ion channels down its concentration gradient so helping to maintain [Ca^2+^]_cyt_ at raised levels. Ca^2+^ released from the dense tubular system is ineffective in eliciting dense granule secretion due to the high Ca^2+^ buffering capacity of the cytosol, which slows the diffusion of Ca^2+^ through the cytosol and reduces the number of unbound Ca^2+^ ions reaching the granule secretory apparatus. Calcium recycling may allow Ca^2+^ to travel relatively unimpeded through the OCS, or create a Ca^2+^ hotspot around Ca^2+^-permeable ion channels which is more effective at triggering Ca^2+^-dependent granule secretion.

An analogous cellular architecture exists in platelets, with the OCS and dense tubular system (DTS; the equivalent of endoplasmic reticulum) being intimately intertwined to form membrane complexes (White 1972). The OCS has recently been elegantly visualized in 3D reconstructions, confirming the close association of this structure with the DTS (van Nispen tot Pannerden et al. 2010). The membrane complexes would provide an appropriate nanojunction for controlling the spread of Ca^2+^ released from the intracellular stores in the DTS, restricting its entry into the bulk cytosol. This ability to control the spatial spread of Ca^2+^ signals would be particular useful in a platelet which when unstimulated is smaller (2–3 μm in diameter) than a Ca^2+^ puff elicited by the opening of a small group of IP_3_ receptors (5 μm; Niggli and Shirokova 2007). In the absence of any control mechanism, platelets would be unable to selectively activate the different Ca^2+^-sensitive processes that are involved in triggering thrombus formation. Yet, platelets can differentially activate these processes in response to weak (e.g., ATP and serotonin) or strong (e.g., thrombin and collagen) agonists, suggesting that some such mechanism must exist. Previous studies have demonstrated the presence of a localized Ca^2+^ gradient in single platelets that would be consistent with some structure controlling the spread of platelet Ca^2+^ signals (Ariyoshi and Salzman 1996). Ca^2+^ released from the DTS may assist in spreading Ca^2+^ through the platelet cytosol by its removal via the NCX from a restricted cytosolic region into the OCS from where it can reenter the cytosol at a site where it can trigger additional Ca^2+^ release from the intracellular stores by Ca^2+^-induced Ca^2+^ release as well as directly maintain the thrombin-evoked rise in [Ca^2+^]_cyt_. These two mechanisms together help potentiate the thrombin-evoked rise in [Ca^2+^]_cyt_ and therefore more rapidly trigger dense granule secretion (Fig. 18). This model is consistent with our images of the pericellular Ca^2+^ signal created by single platelets which suggest its creation within the OCS. Furthermore, the finding that pericellular Ca^2+^ signals are created at specific microdomains within the cell would also be consistent with their generation at the membrane complex. If this model was correct, it would be expected that a macromolecular complex containing both the IP_3_ receptors and Ca^2+^ removal mechanisms might be discovered in this location. Indeed previous work has shown the preferential localization of the plasma membrane Ca^2+^-ATPase to the OCS (Cutler et al. 1980; Herbener and Dean 1988). Although no such data exist for the NCX, we would also expect a tight linkage between the NCX and IP_3_ receptor. Through quantitatively analyzing the Ca^2+^ removal data, we have been able to provide some initial evidence to support this concept. However, the quantitative analysis shows that the only way to account for the fast rate of Ca^2+^ removal measured from the platelet requires the NCX to be exposed to significantly higher Ca^2+^ concentrations than the mean [Ca^2+^]_cyt_ measured by Fura-2. This is consistent with the NCX being colocalized at the membrane complex such that Ca^2+^ released via the IP_3_ receptor creates a microdomain of high Ca^2+^ which drives a rapid turnover rate of the NCX. Although an interesting piece of supportive data, future experiments to show the presence of both Ca^2+^ signaling proteins at this location in the platelet are needed to confirm this hypothesis.

**Figure 19:**
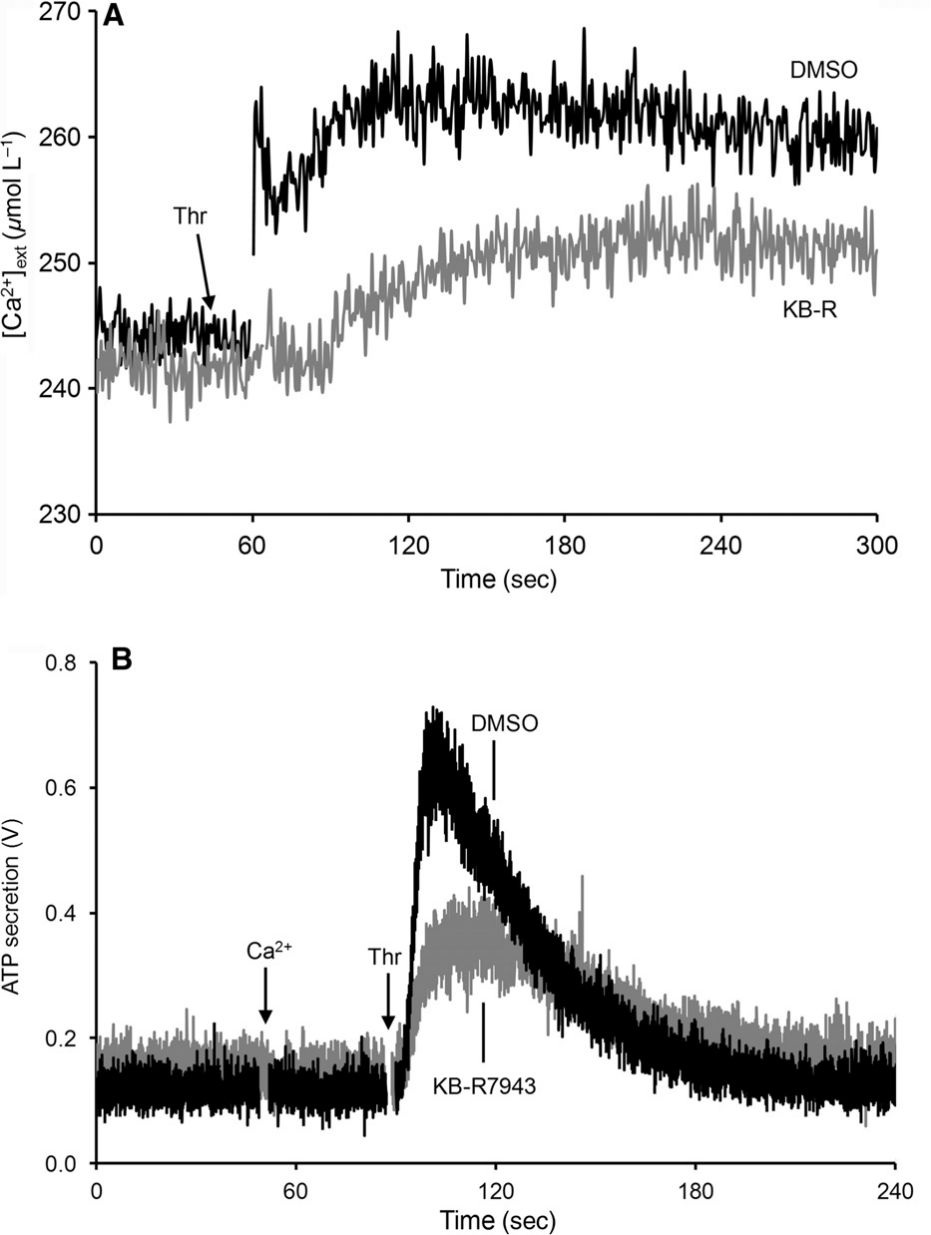
NCX inhibition inhibits thrombin-evoked rises in [Ca^2+^]_ext_ and dense granule secretion elicited in platelets suspended in HBS with a near-physiological extracellular Ca^2+^ concentration. (A) Platelets suspended in a supplemented HBS containing 300 μmol L^−1^ CaCl_2_ and 5 μmol L^−1^ Rhod-5N were preincubated with either 50 μmol L^−1^ KB-R7943, or its vehicle, DMSO, for 2 min at 37°C. Platelets were subsequently stimulated by addition of 0.5 U mL^−1^ thrombin. (B) Human platelets suspended in supplemented HBS were preincubated with either 50 μmol L^−1^ KB-R7943, or its vehicle, DMSO, for 2 min at 37°C. Extracellular Ca^2+^ was raised to 1 mmol L^−1^ by addition of 800 μmol L^−1^ CaCl_2_, cells were then stimulated with 0.5 U mL^−1^ thrombin. ATP secretion was monitored with luciferin–luciferase.

Finally, our data largely concern investigations conducted in Ca^2+^-free media, so there remains a significant question as the physiological relevance of a pericellular recycling system. There is a need to overcome the difficulty of visualizing pericellular Ca^2+^ signals when platelets are suspended in extracellular Ca^2+^ concentrations that will saturate pericellular and extracellular Ca^2+^ indicators. Preliminary studies using the low-affinity Ca^2+^ indicator Rhod-5N have shown that it is possible to monitor changes in [Ca^2+^]_ext_ in the presence of near-physiological extracellular Ca^2+^ concentrations and that events such as reduced thrombin-evoked Ca^2+^ signaling and dense granule secretion following NCX inhibition are observed under these conditions (Fig. 19). As NCX inhibition and other maneuvres designed to interfere with Ca^2+^ recycling affect dense granule secretion, and the NCX inhibitor KB-R7943 (50 μmol L^−1^) reduces the both the initial rate and maximal extent of platelet aggregation (25.3 ± 13.3% and 88.6 ± 2.1% of control, respectively; both *n* = 5, *P* < 0.05, data not shown), our data suggest that Ca^2+^ recycling plays a physiological role in platelet function.

Our data suggest that creating compounds that could disrupt the accumulation of Ca^2+^ in the pericellular region of the platelet might provide a novel method for creating antithrombotic drugs. If this could be achieved specifically it might be possible to reduce platelet dense granule secretion and thus indirectly interfere with autocrine ADP signaling, a factor known to be important for thrombus growth (Nesbitt et al. 2003). Clinical trials have been conducted in stroke patients with a lipophilic divalent cation chelator, DP-b99, designed to chelate divalent cations only in the vicinity of plasma membranes (Diener et al. 2008). Although DP-b99 was trialed as a neuroprotectant, it would be interesting to investigate whether this compound can affect platelet function by interfering with the accumulation of pericellular Ca^2+^.

## Supporting Information

**Video S1.** 3D reconstruction of confocal microscopy data showing the distribution of FFP-18 in human platelets. Note the presence of low-level labelling widely distributed throughout the cell, with a hotspot of high fluorescence observed in an eccentric location within every platelet.

## References

[b1] Alonso MT, Sanchez A, Garcia-Sancho J (1989). Effects of sodium removal on calcium mobilization and dense granule secretion induced by thrombin in human platelets. Biochim. Biophys. Acta.

[b2] Alonso MT, Alvarez J, Montero M, Sanchez A, García-Sancho J (1991). Agonist-induced Ca^2+^ influx into human platelets is secondary to the emptying of intracellular Ca^2+^ stores. Biochem. J.

[b3] Ariyoshi H, Salzman EW (1996). Association of localized Ca^2+^ gradients with redistribution of glycoprotein IIb-IIIa and F-actin in activated human blood platelets. Arterioscler. Thromb. Vasc. Biol.

[b4] Blatter LA, Niggli E (1998). Confocal near-membrane detection of calcium in cardiac myocytes. Cell Calcium.

[b5] Blaustein MP, Lederer W (1999). Sodium/Calcium exchange: its physiological implications. Physiol. Rev.

[b6] Bolsover SR (1986). Two components of voltage-dependent calcium influx in mouse neuroblastoma cells: measurement with Arsenazo III. J. Gen. Physiol.

[b7] Brass LF (1984). The effect of Na^+^ on Ca^2+^ homeostasis in unstimulated platelets. J. Biol. Chem.

[b8] Brass LF, Shattil SJ (1982). Changes in surface-bound and exchangeable calcium during platelet activation. J. Biol. Chem.

[b9] Burkhart JM, Vaudel M, Gambaryan S, Radau S, Walter U, Martens L (2012). The first comprehensive and quantitative analysis of human platelet protein composition allows the comparative analysis of structural and functional pathways. Blood.

[b10] Cutler LS, Feinstein MB, Christian CP (1980). Cytochemical localization of ouabain-sensitive (K+)-dependent p-nitrophenyl phosphatase (transport ATPase) in human blood platelets. J. Histochem. Cytochem.

[b11] Deisseroth K, Bito H, Tsien RW (1996). Signalling from synapse to nucleus: postsynaptic CREB phosphorylation during multiple forms of hippocampal synaptic plasticity. Neuron.

[b12] Diener HC, Schneider D, Lampl Y, Bornstein NM, Kozak A, Rosenberg G (2008). DP-b99, a membrane-activated metal ion chelator, as neuroprotective therapy in ischemic stroke. Stroke.

[b13] DiPolo R, Beaugé L (1996). Sodium/Calcium Exchanger: influence of metabolic regulations on ion carrier interactions. Physiol. Rev.

[b14] Etter EF, Minta A, Poenie M, Fay FS (1996). Near-membrane [Ca^2+^] transients resolved using the Ca^2+^ indicator FFP18. Proc. Natl Acad. Sci. USA.

[b15] Fromjovich MM, Milton JG (1982). Human platelet size, shape and related functions in health and disease. Physiol. Rev.

[b16] Fung CYE, Cendana C, Farndale RW, Mahaut-Smith MP (2007). Primary and secondary agonists can use P2X_1_ receptors as a major pathway to increase intracellular Ca^2+^ in the human platelet. J. Thromb. Haemost.

[b17] van Gorp RM, Feijge MA, Vuist WM, Rook MB, Heemskerk JWM (2002). Irregular spiking in free calcium concentration in single, human platelets. Regulation by modulation of the inositol trisphosphate receptors. Eur. J. Biochem.

[b18] Grynkiewicz G, Poenie M, Tsien RY (1985). A new generation of Ca^2+^ indicators with greatly improved fluorescence properties. J. Biol. Chem.

[b19] Hallam TJ, Rink TJ (1985). Agonists stimulate divalent cation channels in the plasma membrane of human platelets. FEBS Lett.

[b20] Harper AGS, Sage SO (2007). A key role for reverse Na^+^/Ca^2+^ exchange influenced by the actin cytoskeleton in store-operated Ca^2+^ entry in human platelets: evidence against the de novo conformational coupling hypothesis. Cell Calcium.

[b21] Harper AGS, Brownlow SL, Sage SO (2009a). A role for TRPV1 in agonist-evoked activation of human platelets. J. Thromb. Haemost.

[b22] Harper AGS, Mason MJ, Sage SO (2009b). A key role for dense granule secretion in potentiation of the Ca^2+^ signal arising from store-operated calcium entry in human platelets. Cell Calcium.

[b23] Harper MT, Mason MJ, Sage SO, Harper AGS (2010). Phorbol ester-evoked Ca^2+^signaling in human platelets is via autocrine activation of P_2X1_ receptors, not a novel non-capacitative Ca^2+^ entry. J. Thromb. Haemost.

[b24] Heemskerk JWM, Willems GM, Rook MB, Sage SO (2001). Ragged spiking of free calcium in ADP-stimulated human platelets: regulation of puff-like calcium signals in vitro and ex vivo. J. Physiol.

[b25] Herbener GH, Dean WL (1988). Immunocytochemical localization of the Ca^2+^-ATPase polypeptide in human platelets. Biochem. Biophys. Res. Commun.

[b26] Holmsen H, Weiss HJ (1979). Secretable storage pools in platelets. Annu. Rev. Med.

[b27] Hunyady L, Sarkadi B, Spät EJ, Cragoe A, Gárdos G (1987). Activation of sodium-proton exchange is not a prerequisite for Ca^2+^ mobilization and aggregation in human platelets. FEBS Lett.

[b28] Iwamoto T, Shigekawa M (1998). Differential inhibition of Na^+^/Ca^2+^ exchanger isoforms by divalent cations and isothiourea derivative. Am. J. Physiol.

[b29] Jones S, Solomon A, Sanz-Rosa D, Moore C, Holbrook L, Cartwright EJ (2010). The plasma membrane calcium ATPase modulates calcium homeostasis, intracellular signaling events and function in platelets. J. Thromb. Haemost.

[b30] Jy W, Haynes DH (1987). Thrombin-induced calcium movements in platelet activation. Biochim. Biophys. Acta.

[b31] Lages B, Weiss HJ (1999). Secreted dense granule adenine nucleotides promote calcium influx and the maintenance of elevated cytosolic calcium levels in stimulated human platelets. Thromb. Haemost.

[b32] Lewandrowski U, Wortelkamp S, Lohrig K, Zahedi RP, Wolters DA, Walter U (2009). Platelet membrane proteomics: a novel repository for functional research. Blood.

[b33] MacIntyre DE, Rink TJ (1982). The role of platelet membrane potential in the initiation of aggregation. Thromb. Haemost.

[b34] Mahaut-Smith MP (1995). Calcium-activated potassium channels in human platelets. J. Physiol.

[b35] Mahaut-Smith MP, Rink TJ, Collins SC, Sage SO (1990). Voltage-gated potassium channels and the control of membrane potential in human platelets. J. Physiol.

[b36] Merritt JE, Armstrong WP, Benham CD, Hallam TJ, Jacob R, Jaxa-Chamiec A (1990). SK&F 96365, a novel inhibitor of receptor-mediated calcium entry. Biochem. J.

[b37] Mihalyi E (2004). A review of some unusual effects of calcium binding to fibrinogen. Biophys. Chem.

[b38] Mogami H, Gardner J, Gerasimenko OV, Camello P, Petersen OH, Tepikin AV (1999). Calcium binding capacity of the cytoplasm and endoplasmic reticulum of mouse pancreatic acinar cells. J. Physiol.

[b39] Nazer MA, van Breemen C (1998). Functional linkage of Na^+^-Ca^2+^ exchange and sarcoplasmic reticulum Ca^2+^ release mediates Ca^2+^ cycling in vascular smooth muscle. Cell Calcium.

[b40] Neco P, Rose B, Hyunh N, Zhang R, Bridge JHB, Philipson KD (2010). Sodium-calcium exchange is essential for effective triggering calcium release in mouse heart. Biophys. J.

[b41] Nesbitt WS, Giuliano S, Kulkarni S, Dopheide SM, Harper IS, Jackson SP (2003). Intercellular calcium communication regulates platelet aggregation and thrombus growth. J. Cell Biol.

[b42] Niggli E, Shirokova N (2007). A guide to sparkology: the taxonomy of elementary cellular Ca^2+^ signalling events. Cell Calcium.

[b43] van Nispen tot Pannerden H, Geerts F, de Haas W, Posthuma G, Heijnen S, van Dijk HFG (2010). The platelet interior revisited: electron tomography reveals tubular α-granule subtypes. Blood.

[b44] Orchard CH, Pasek M, Brette F (2009). The role of mammalian cardiac t-tubules in excitation–contraction coupling: experimental and computational approaches. Exp. Physiol.

[b45] Petersen OH, Verkhratsky A (2007). Endoplasmic reticulum calcium tunnels integrate signalling in polarized cells. Cell Calcium.

[b46] Pethig R, Kuhn M, Payne R, Adler E, Chen TH, Jaffe LF (1989). On the dissociation constants of BAPTA-type calcium buffers. Cell Calcium.

[b47] Pipili E (1985). Platelet membrane potential: simultaneous measurement of DiSC3(5) fluorescence and optical density. Thromb. Haemost.

[b48] Prakriya M, Lewis RS (2003). CRAC channels: activation, permeation and the search for a molecular identity. Cell Calcium.

[b49] Purvis JE, Chatterjee MS, Brass LF, Diamond SL (2008). A molecular signalling model of platelet phosphoinositide and calcium regulation during homeostasis and P_2Y1_ activation. Blood.

[b50] Rengasamy A, Soura S, Feinberg H (1987). Platelet Ca^2+^ homeostasis: Na^+^-Ca^2+^ exchange in plasma membrane vesicles. Thromb. Haemost.

[b51] Rink TJ, Sage SO (1990). Calcium signalling in human platelets. Annu. Rev. Physiol.

[b52] Rittenhouse SE, Sasson JP (1985). Mass changes in myoinositol trisphosphate in human platelets stimulated by thrombin. J. Biol. Chem.

[b53] Roberts DE, McNicol A, Bose R (2004). Mechanism of collagen activation in human platelets. J. Biol. Chem.

[b54] Roberts DE, Matsuda T, Bose R (2012). Molecular and functional characterization of the human platelet Na^+^/Ca^2+^ exchangers. Br. J. Pharmacol.

[b55] Rosado JA, Meijer EM, Hamulyak K, Novakova I, Heemskerk JW, Sage SO (2001). Fibrinogen binding to the integrin alpha(IIb)beta(3) modulates store-mediated calcium entry in human platelets. Blood.

[b155] Ruiz FA, Lea CR, Oldfield E, Docampo R (2004). Human platelet dense granules contain polyphosphate and are similar to acidocalcisomes of bacteria and unicellular eukaryotes. J. Biol. Chem.

[b56] Sage SO, Rink TJ (1987). The kinetics of changes in intracellular calcium concentration in fura-2-loaded human platelets. J. Biol. Chem.

[b57] Sage SO, Pugh N, Mason MJ, Harper AGS (2011). Monitoring the intracellular Ca^2+^ concentration in agonist-stimulated, intact human platelets using Fluo-5N. J. Thromb. Haemost.

[b58] Sanchez A, Alonso MT, Collazos JM (1988). Thrombin-induced changes of intracellular [Ca^2+^] and pH in human platelets. Cytoplasmic alkalinisation is not a prerequisite for calcium mobilization. Biochim. Biophys. Acta.

[b59] Sato T, Herman L, Chandler JA, Stracher A, Detwiler TC (1975). Localisation of a thrombin-sensitive calcium pool in platelets. J. Histiochem. Cytochem.

[b60] Schaeffer J, Blaustein MP (1989). Platelet-free calcium concentrations measured with fura-2 are influenced by the transmembrane sodium gradient. Cell Calcium.

[b61] Schwaller B (2010). Cytosolic Ca^2+^ buffers. Cold Spring Harb. Perspect. Biol.

[b62] Siffert W, Akkerman JWN (1987). Intracellular pH and cytoplasmic free Ca^2+^. Nature.

[b64] Somasunduram B, Mason MJ, Mahaut-Smith MP (1997). Thrombin dependent calcium signalling in single human erythroleukaemia cells. J. Physiol.

[b65] Stamouli V, Vakirtzi-Lemonias C, Siffert W (1993). Thrombin and NaF, but not epinephrine, raise cytosolic free Na^+^ in human platelets. Biochim. Biophys. Acta.

[b66] Steiner M (1986). Role of glycosaminoglycans in calcium metabolism of human platelets. Biochim. Biophys. Acta.

[b67] Stern MD (1992). Buffering of calcium in the vicinity of a channel pore. Cell Calcium.

[b68] Tang Q, Jin M-W, Xiang J-Z, Dong M-Q, Sun H-Y, Lau C-P (2007). The membrane permeable calcium chelator BAPTA-AM directly blocks human ether ether a-go-go-related gene potassium channels stably expressed in HEK 293 cells. Biochem. Pharmacol.

[b69] Urbano FW, Buño W (1998). BAPTA-AM blocks both voltage-gated and Ca^2+^-activated K^+^ currents in cultured bovine chromaffin cells. NeuroReport.

[b70] White JG (1972). Interaction of membrane systems in blood platelets. Am. J. Pathol.

